# Supplementary material for: Fast assembling of neuron fragments in serial 3D sections
Source: Brain Inform. 2017 Apr 1;4(3):183–6. doi: 10.1007/s40708-017-0063-9 (PMC5563299; doi:10.1007/s40708-017-0063-9)
Supplement: Supplementary file 1 — Supplementary material 1 (DOCX 5365 kb) [file 40708_2017_63_MOESM1_ESM.docx]

Supplemental Materials of “Fast Assembling of Neuron Fragments in Serial 3D Sections”

*Hanbo Chen^1,2^, Daniel M. Iascone^3^, Nuno Macarico da Costa^1^, Ed S. Lein^1^, Tianming Liu^2^, Hanchuan Peng^1,*^*

^1^Allen Institute for Brain Science, Seattle, WA, USA. ^2^Cortical Architecture Imaging and Discovery Laboratory, Department of Computer Science and Bioimaging Research Center, The University of Georgia, Athens, GA, USA; ^3^Department of Neuroscience, Columbia University, New York, NY, USA.
* Corresponding author.

**Table of Content**

[1 Related Works 3](#_Toc466497308)

[2 Match, Align, and Stitch Reconstruction 4](#_Toc466497309)

[2.1 Neuron Reconstruction 5](#_Toc466497310)

[2.2 Identify Border-tips 5](#_Toc466497311)

[2.2.1 Reconstruction gaps 6](#_Toc466497312)

[2.2.2 Spine tips 6](#_Toc466497313)

[2.2.3 Fragments of background noise 7](#_Toc466497314)

[2.3 Match Border-tips and Estimate Alignment 7](#_Toc466497315)

[2.3.1 Triangle Match 8](#_Toc466497316)

[2.3.2 Border-tips Matching Probability 9](#_Toc466497317)

[2.3.3 Estimate Alignment 9](#_Toc466497318)

[2.4 Stitch Border-tips 10](#_Toc466497319)

[3 Interactive User Interface 10](#_Toc466497320)

[3.1 Stitch Reconstructions 11](#_Toc466497321)

[3.1.1 A. Automatically match and align reconstructions (Figure S 6, Video S 1) 11](#_Toc466497322)

[3.1.2 B. Visually check and stitch matched reconstructions (Figure S 7, Video S 2-3) 12](#_Toc466497323)

[3.1.3 C. Manually correct matching result (Figure S 8, Video S 4) 13](#_Toc466497324)

[3.2 Select Border-tips 14](#_Toc466497325)

[3.2.1 D. Select border-tips (Figure S 9) 14](#_Toc466497326)

[3.2.2 E. Visually inspect border-tips (Figure S 9) 14](#_Toc466497327)

[4 Results 16](#_Toc466497328)

[4.1 Validation on Ground Truth Data 16](#_Toc466497329)

[4.2 Large Scale Neuron Image in Mammalian Brain 17](#_Toc466497330)

[4.3 Simulated Testing Data 23](#_Toc466497331)

[5 Comparison 26](#_Toc466497332)

[Reference 29](#_Toc466497333)

**Table of Tables**

[Table S 1. A survey of previous works related to neuron sections alignment. 3](#_Toc466476562)

[Table S 2. Information of data applied for stitching test cases. 17](#_Toc466476563)

[Table S 3. Stitching results between sections based on the live stitching module. 18](#_Toc466476564)

**Table of Figures**

[Figure S 1. The software architecture of NeuronStitcher. 5](#_Toc466476587)

[Figure S 2. Examples of 3 types of tips that should not be considered as border-tips 6](#_Toc466476588)

[Figure S 3. Decision tree to determine whether a tip is *spine tip* or *neurite tip*. 7](#_Toc466476589)

[Figure S 4. Illustration of the rotation invariant features of the triangle to match. 8](#_Toc466476590)

[Figure S 5. Illustration of adjusting branches to stitch matched border-tips. 10](#_Toc466476591)

[Figure S 6. Illustration of automatically matched and aligned reconstructions. 12](#_Toc466476592)

[Figure S 7. Illustration of visual check and stitch matched reconstructions. 13](#_Toc466476593)

[Figure S 8. Illustration of manually corrected matching result. 14](#_Toc466476594)

[Figure S 9. Illustration of visual inspection and semi-automatic selection of border-tips. 15](#_Toc466476595)

[Figure S 10. A visual comparison between the reconstruction from the tissue before sectioning and the stitched reconstruction from sectioned tissues 17](#_Toc466476596)

[Figure S 11. Visualization of stitching results of dataset 1 from different views. 19](#_Toc466476597)

[Figure S 12. Visualization of complete neuron reconstruction of dataset 1 after stitching 20](#_Toc466476598)

[Figure S 13. Visualization of stitching results of dataset 2 from different views. 21](#_Toc466476599)

[Figure S 14. Visualization of the complete neuron reconstruction of dataset 2 after stitching. 21](#_Toc466476600)

[Figure S 15. Visualization of stitching results of dataset 3 from different views 22](#_Toc466476601)

[Figure S 16. Visualization of the complete neuron reconstruction of dataset 3 after stitching. 23](#_Toc466476602)

[Figure S 17. Illustration of the generation of simulated data from experimental data 23](#_Toc466476603)

[Figure S 18. The performance of automatic matching module on simulated data 24](#_Toc466476604)

[Figure S 19. The performance of Filament Editor in matching and aligning neuron fragments 28](#_Toc466476605)

Supplementary videos, software packages, and testing data are available at:

*http://cobweb.cs.uga.edu/~hanbo/4press/neuronStitcher/*

# Related Works

Digital reconstruction of neurons from very large three-dimensional (3D) brain images is crucial for modern neuroscience^1–3^. Despite recent advances in neuron labeling, brain clearing, and high-resolution 3D tissue-imaging^4,5^ to study mammalian brains, many neuroscientists still rely on physical sectioning of brains followed by imaging with confocal or two-photon microscopy. The resulting image data over many serial sections are then stacked and aligned individually to generate a complete image volume, from which neurons are reconstructed and quantified. Dendrites and axons that cross an imaged volume and were severed at the section boundaries then need to be stitched. This is a crucial step and a bottleneck for the proper reconstruction of the topology of the dendritic and axonal trees, which is particularly difficult given the density of local axonal arborization. It is extremely labor-intensive to stitch neuron segments manually over multiple sections^6,7^. Automated methods can, therefore, provide a significant increase in the throughput of neuron reconstruction.

However, this is a non-trivial task for automatic algorithms as there could be missing tissue as well as distortions during sectioning, making stitching of neuronal segments across multiple sections much more challenging than stitching overlapping tiles within single sections^8,9^. Moreover, unlike stitching serial 2D images from serial electron microscopy (EM)^10,11^, neurons imaged with confocal and two-photon microscopes are often sparsely distributed throughout the 3D image stacks, making it difficult to use other stitching tools such as TrakEM2^11^.

In previous works, a couple of automatic or semiautomatic computational frameworks have been proposed to solve this problem. We surveyed related works and summarized them in Table S 1. Though these works applied different algorithms and had different designs, most of them performed match and alignment on traced neuron reconstructions and the key procedure includes following 3 steps: (1) identify landmarks such as neuron fragment termination on the sectioning plane; (2) match landmarks; (3) estimate alignment based on matched landmarks. Some of the early works performed these steps manually or semi-automatically. In recent works, matching algorithms were adopted to automate the whole process.

In our survey, we found these previous works have following limitations. (1) Most tools are not readily available in their Open Source form. So far, the only available tool for such task is Filament Editor^12,13^ which is a plugin function of a commercial software Amira. (2) Their performance has not been comprehensively evaluated. Most of the matching algorithm requires the selection of a set of parameters. The impact of these parameters on the performance of the algorithm has been rarely discussed in results. (3) The landmark selection method is not well designed to handle the noise in real data. Most methods simply picked all the neuron trace terminations close to the sectioning plane as landmarks. This approach will also pick noise patterns generated by the reconstruction and thus suppress the accuracy and the speed of matching algorithms.

Table S 1. A survey of previous works related to neuron sections alignment.

| Method | Matching Algorithm | Performance | Robustness | Availability |
| --- | --- | --- | --- | --- |
| Weber et al, 2014^13^ | Estimate initial and final match by large cliques in distance compatibility graph with angle information.  Refine match by Markov random field and elastic alignment. | Tested on electron tomogram sections, and compared against manually produced “gold standard”. | The tool is designed for dense microtubule across serial electron tomograms. | Available as an extension of commercial software Amira. |
| Dercksen et al, 2009^14^  Dercksen et al, 2014^12^ | Estimate initial match and alignment by distance compatibility graph. Refine match by automatic greedy point match. (No branch direction information included) | Tested on neuron fragment and microtubule datasets: Running time < 8.5s/pair; Average distance to manual alignment is 25.2µm. | Method failed (mentioned by authors) on 3 neuron slices where there are too few points and on 2 microtubule slices without clear explanations. | Available as an extension of commercial software Amira. |
| Hogrebe et al, 2011^15^ | Landmark matching based on neighborhood and angles. | Tested on small slices of neuron tissues. | Landmark matching based on neighborhood and angles. | Tested on small slices of neuron tissues. |
| Luzzati et al, 2011^7^ | Requires manual selection of matching points to estimate transformation. | Tested on neuron slices with different imaging parameters.  Extremely time-consuming due to manual selection.  No accuracy measurement. | Rely on manual operations. | Need to switch between 6 free software packages: Reconstruct, Fiji, Vaa3D, VIAS, Neuronstudio, Neuromatic. |
| Oberlaender et al, 2007^6^ | Manually spliced. | 12 slices of neuron reconstruction fragments were aligned and spliced. | NA | Manually spliced using commercial software Neurolucida. |
| ImageJ/TrakEM2^10^ | Not applicable to our application cases | | | |

# Match, Align, and Stitch Reconstruction

The goal of NeuronStitcher is to first align and connect neuron fragments across sections and then infer a complete neuron reconstruction. In practice, the order of sections is usually known. The sections can be matched and aligned by shifting and rotating reconstructions in parallel to the sectioning plane. For a set of input neuron fragments, the task can be defined by three major steps: (1) identify severed neurites candidates which will be matched and connected later on; (2) match severed neurites and align reconstructions from different sections; (3) connect matched neurites. The whole pipeline is outlined in blue in Figure S 1. Each step is elaborated in this section.


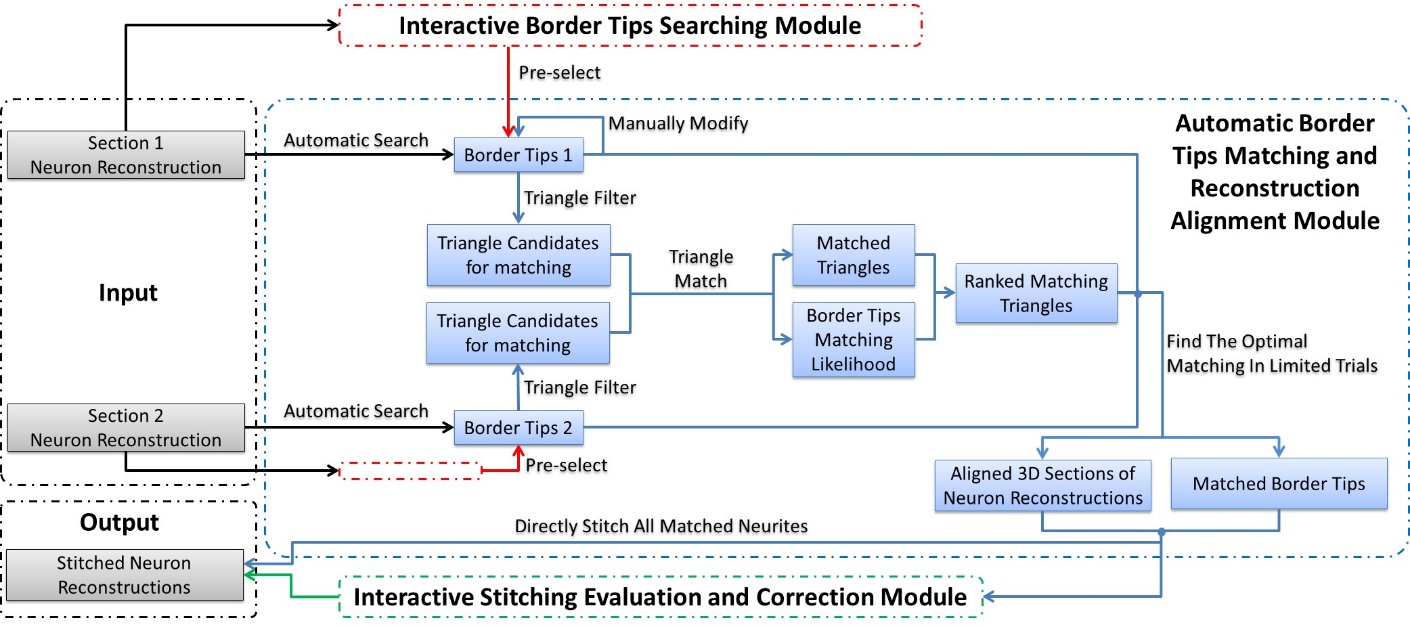


Figure S 1. The software architecture of NeuronStitcher. The software takes a set of neuron-reconstruction fragments as input and outputs the stitched neuron reconstruction. It includes an automatic module to match, align, and stitch neuron reconstructions, as well as two interactive operation modules which allow users to visually inspect and manually correct results. The computational pipeline of automatically stitching module is outlined in blue.

## Neuron Reconstruction

A neuron reconstruction typically encodes the morphology of the neuron using a tree structure. Usually, connected vertices are taken as basic units in a reconstruction file. For each vertex, its coordinate, parent vertex, and radius are recorded. In previous studies, many automatic and semiautomatic tools have been proposed to reconstruct neuron morphology from microscopy images. One may refer to the review paper by Duncan and Ascoli^16^ for a survey of neuron reconstruction methods. In this paper, the initial reconstructions are obtained based on the built-in tools of publicly available Vaa3D software^17,18^. Nevertheless, our NeuronStitcher tool is generally applicable to neuron reconstructions produced by other tools.

Notably, since the image resolution in the depth direction (z-axis) is usually lower than those of plane directions (x-axis and y-axis), we rescale the reconstructed neurite-fragments to isotropic “resolution” before analysis.

## Identify Border-tips

We defined the severed neurites at the section plane as border-tips. Intuitively, the terminal branches identified near the section plane can be taken as border-tips. However, there are also other sources of terminal branches near the section plane and some of them can be excluded by a simple method to increase accuracy and efficiency of the matching algorithm. Based on our observation, 3 types of tips that should be excluded were identified (Figure S 2) – (1) reconstruction gaps, (2) spine, and (3) fragments of background noise. We explained those tips with details and introduced our strategy to automatically exclude them below. This border-tips searching framework was embedded in the automatic matching and alignment module.


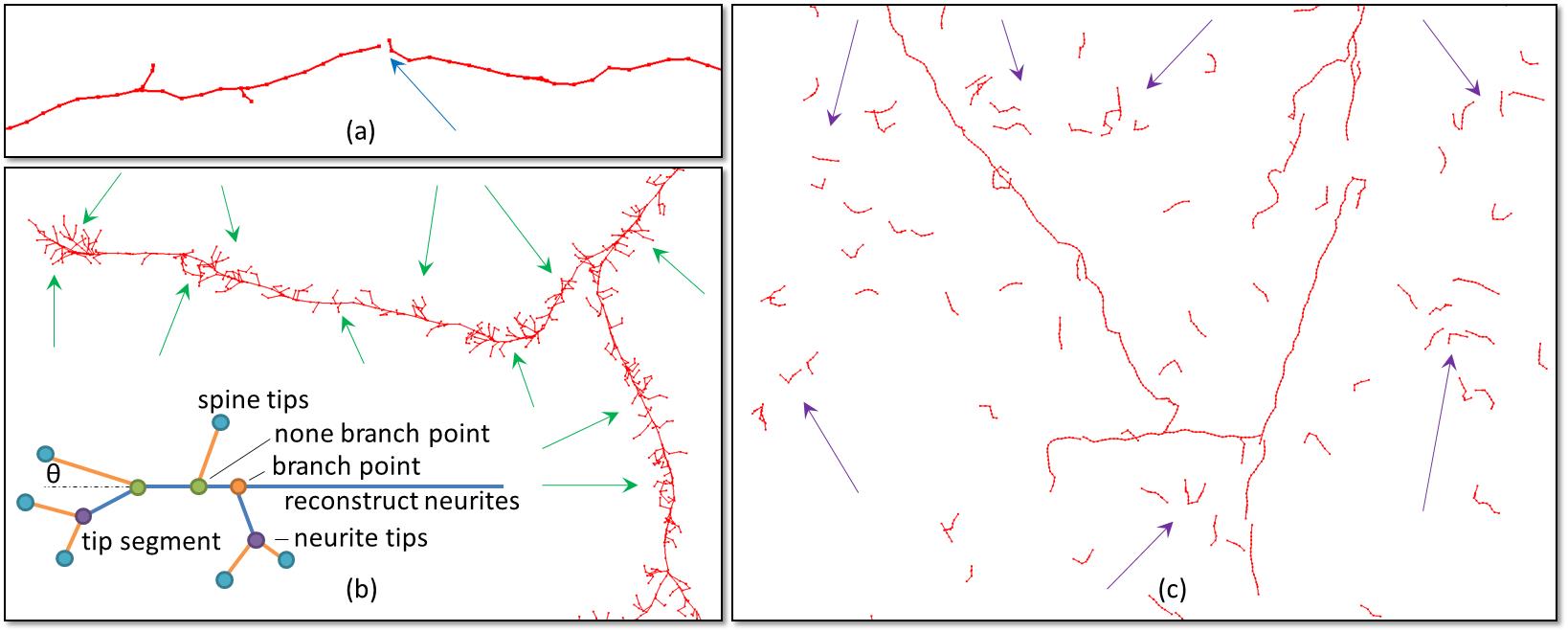


Figure S 2. Examples of 3 types of tips that should not be considered as border-tips. (a) An example of a gap in the reconstruction as highlighted by the blue arrow. (b) Examples of spine tips as highlighted by green arrows. (c) Examples of fragments of background noise as highlighted by purple arrows.

### Reconstruction gaps

Some reconstruction algorithms generate a gap between segments as shown in (Figure S 2 (a)). Usually, the gap can be erased and the segments are connected after post-processing to build complete neuron tree structure. However, if the gap was close to the section plane, the introduced neurite tips can be identified as border-tips and be taken as candidates to be matched and connected with the border-tips from the adjacent sections.

The tips introduced by reconstruction gaps are usually generated in pairs, where they are close but not connected to each other. Thus, to filter them, a gap size threshold is defined such that, if a set of border-tips are 1) close to each other (distance < gap threshold), and 2) not connected (no path exists between them) in current reconstruction, they will be identified as a reconstruction gap and excluded from further analysis.

### Spine tips

Some of the neuron reconstruction algorithms also trace spine structures during reconstruction (Figure S 2 (b)). If we take the spine structures close to section plane as border-tips, they will result in a lot of noise. We, therefore, need an efficient solution to eliminate the tips from spine structures and find the tips of the severed neurites for matching. To better explain our solution, some terminology is defined below.

*Spine tip*: Tip on reconstructed spine structure (blue dots in Figure S 2 (b)).

*Tip segment*: The section of the reconstruction from the tip to its nearest fork (orange lines in Figure S 2 (b)).

*Tip segment turning angle*: The angle between a tip segment and its parent segment (θ in Figure S 2 (b)).

*Neurite tip*: The tips of the neurite that could be taken as border-tips for matching (purple dots in Figure S 2 (b)).

Reconstructed spines are a relatively thin, short segments that are perpendicular to neurites. A tip is defined as a *spine tip* if it meets three criteria: (1) tip segment is short, (2) turning angle is relatively large, and (3) radius is small. For each tip, its identity as a *neurite tip* or *spine tip* is determined based on a decision tree shown in Figure S 3. The size, turning angle, and radius of the tip segment will be examined and the tip will be classified as a *spine tip* if and only if all three criteria were met. In addition to the remaining *neurite tips*, the tips that are newly generated by removing the spine segment are defined as neurite tips (purple dots in Figure S 2 (b)).


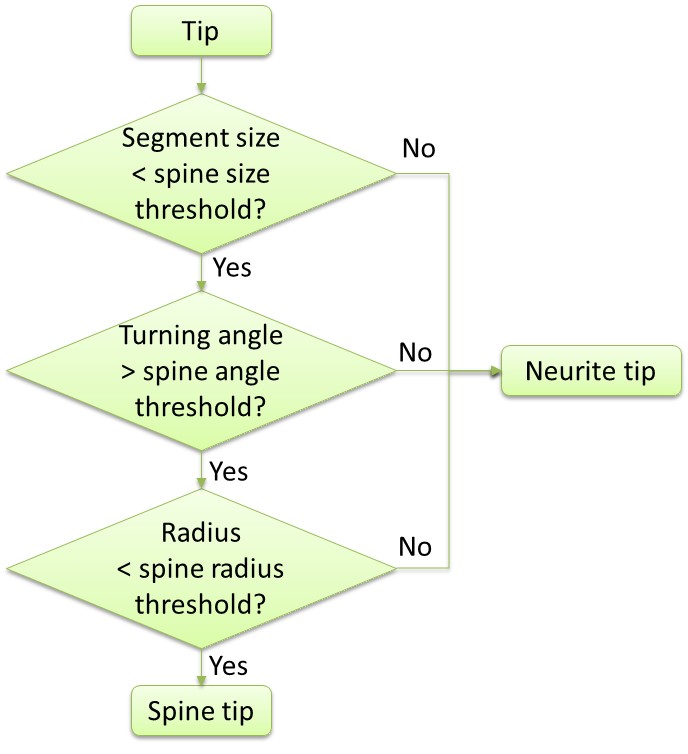


Figure S 3. Decision tree to determine whether a tip is *spine tip* or *neurite tip*.

### Fragments of background noise

Some reconstruction algorithms do not eliminate background noise and thus generate small noise fragments (highlighted by arrows in Figure S 2 (c)). Intuitively, such fragments can be eliminated by looking at the size of each reconstruction fragment and ignoring the tips of small fragments that should not be considered to be border-tips.

Nevertheless, because reconstruction quality and character vary between datasets, image modality, and reconstruction method, it is hard to guarantee that all the border-tips that are automatically identified are true severed neurites. Further, the algorithm can result in a false positive connection when falsely detected border-tip gets matched. With this in mind, we included an interactive border-tips searching module to take advantage of user knowledge in the identification of border-tips. In this module, the automatically identified border-tips are shown together with the raw image and reconstructions. The user can then visually screen through the results and accept, reject, or manually add border-tips. More details are shown in section 3.2.

## Match Border-tips and Estimate Alignment

It is intuitive to match adjacent sections by matching border-tips such that a correct match will allow most severed neurites to be reasonably connected. To achieve this, two factors can be taken into consideration: 1) Geodesic location of border-tips – the neighborhood relation of the paired border-tips should match. 2) The orientation of the branch connected to border-tips – two branches should not be connected when there is a sharp angle between them. In the study of computer vision, affine-invariant features based on triangle shapes have been widely applied to match objects in images and graphs ^19,20^. Because our matching object requires rotation and shifting to be matched and the orientation of the branch connected to border-tips changes during rotation, we adopted ideas from previous work and proposed a set of rotation invariant features to describe neighborhood and branch orientation based on triangles (Section 2.3.1). Based on a matched triangle, the matching probability between border-tips is then defined (Section 2.3.2). By taking the matched border-tip pairs with the highest probability as an initial match, alignment and matching can be estimated and refined iteratively for an optimal alignment (Section 2.3.3). In this section, we will introduce our algorithm to solve the match and alignment problem in two adjacent slices. A and B represent the set of border-tips between these two sections accordingly. Without loss of generality, we assume that A will be fixed and B will be moved and aligned to A.

### Triangle Match

For any combination of 3 border-tips, a triangle can be constructed (for illustration and symbols notation, see Figure S 4). For each triangle, its edges and vertices are sorted by the length of edges such that $\left\| e_{1,2} \right\|\leq\left\| e_{2,3} \right\|\leq\left\| e_{1,3} \right\|$ where *e* is the edge of the triangle. The sorted length of its edges is then applied to describe the geodesic shape of a triangle: *D=*(*d_1,2_*, *d_2,3_*, *d_1,3_*), *d_i,j_*=║*e_i,j_*║. To quantify the orientation of branches, a local coordinate system is defined for each vertex such that the axis from triangle center to the vertex is the x-axis and the norm direction of the triangle plane is the z-axis (Figure S 4). The orientation vector of the branch connected to the border-tip is then transformed into this local coordinate system. Two triangles Δ*_I_,* Δ*_J_* and the corresponding vertices could be matched when the *L_1_* distance between their geodesic shape vector *D* and the angle between branch orientation vectors of corresponding vertices are smaller than threshold defined:

| $\Delta_{I}\cong\Delta_{J},v_{I1}=v_{J1},v_{I2}=v_{J2},v_{I3}=v_{J3}$ | (1) |
| --- | --- |

if and only if:

| $\left\vert D_{I}-D_{J} \right\vert<distance threshold$ | (2) |
| --- | --- |
| $angle\left( \vec{T_{Ik}},\vec{T_{Jk}} \right)<angular threshould(k=1,2,3)$ | (3) |

Notably, since the angle is defined in the local coordinate of each vertex, these proposed features are rotation invariant. Thus, triangles can be matched directly based on these features without alignment.


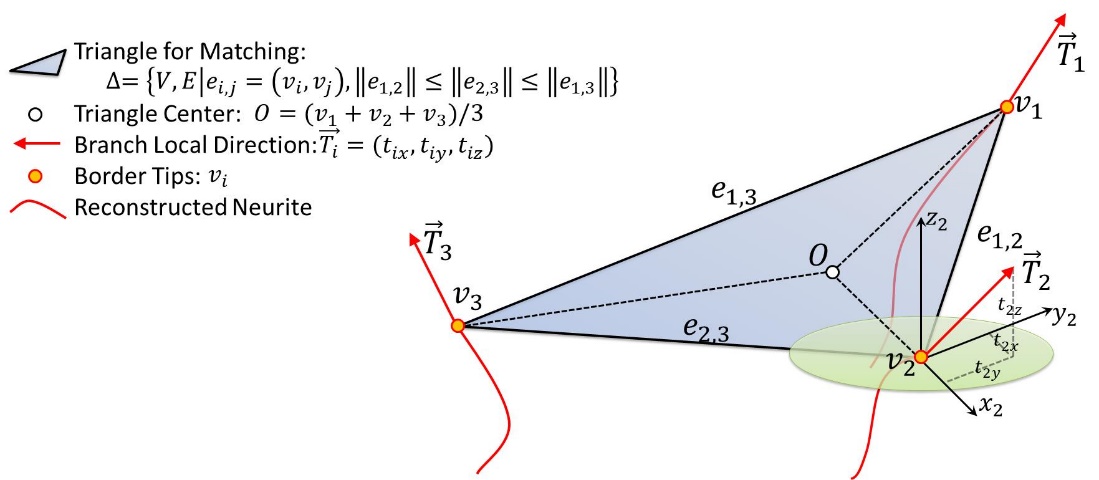


Figure S 4. Illustration of the rotation invariant features of the triangle to match.

### Border-tips Matching Probability

Based on the triangle matching previously proposed, a global search can be performed to pair-wisely match all triangles formed by border-tips. Then, for each pair of border-tips $\left( v_{a},v_{b} \right)$, the number of times they get matched $N_{a,b}$ is accumulated and their matching probability is defined as:

| $P_{a,b}={2N_{a,b}}/\left( \sum_{k\in A} N_{k,b}+\sum_{k\in B} N_{a,k} \right)$ | (4) |
| --- | --- |

For each pair of matched triangles $\left( \Delta_{a},\Delta_{b} \right),a\subset A,b\subset B$, the matching likelihood between them is computed by summing the matching probability between the corresponding vertices. The pair of matched triangles with the highest matching likelihood can be taken as the initial match to estimate alignment.

However, the number of triangles grows cubically with the number of border-tips. If the number of border-tips identified on the section plane of two adjacent sections is *|A|* and *|B|*, the computational complexity for matching is *O*(*|A|*^3^×|B|^3^). This can be extremely time-consuming when *A* and *B* are large. To increase computational efficiency, we found that it is unnecessary to compare all triangles. Specifically, for the purpose of matching corresponding vertices, the matching based on the triangles with anisotropic shape was more reliable than equilateral triangles. Moreover, large triangles offer more global view than small triangles. Based on these observations, we propose a triangle priority score in which only the top triangles with the highest scores are applied to estimating border-tips matching probability. Given a triangle with geodesic shape feature: *D=*(*d_1,2_*, *d_2,3_*, *d_1,3_*), its priority score is simply defined as the difference between the lengths of its longest edge and the shortest edge:

| $Score=d_{1,3}-d_{1,2}$ | (5) |
| --- | --- |

We show in the result section that only matching a few hundred triangles with the highest scores is sufficient to infer accurate results while the computational time required is reduced from minutes to seconds.

### Estimate Alignment

Because adjacent sections are usually imaged separately from mounted sections, each section can be aligned by shifting it in all three directions and rotating it in parallel to the section plane (4 degrees of freedom (4DOF). While there could be other types of movement and distortions between sections, our results on existing data suggested that the 4DOF alignment is sufficiently accurate to generate reasonable results. Such alignment can be easily estimated based on the matched border-tips between sections in the following two steps. 1^st^) Shifting movement will be estimated such that the center of matched border-tips will be the same. 2^nd^) Rotation around the center will be estimated to minimize the distance between matched border-tips.

The pair of matched triangles with the highest matching likelihood is taken as the initial match. After transformation, the border-tips that are close to each other and have similar branch-orientations are matched. Based on updated matching border-tips, a new transformation is estimated. This match-transform procedure is iterated until no more border-tips can be matched. Notably, if the initial match is incorrect, the final alignment will also be wrong. Thus different initial combinations are examined and the final alignment that has the greatest number of matching border-tips is taken as the result.

Notably, the proposed triangle match method requires at least 3 border-tips on both sides of the sectioning plane. When there are less than 3 border-tips identified, we will use a simple greedy approach instead to find the best match such that after alignment, the distance and the angle between matched pairs are minimized.

## Stitch Border-tips

After affine transformation, the matched border-tips are connected to construct a complete neuron. Because slicing causes distortion which leads to systematic errors in imaging and reconstruction procedures, a gap between matching border-tips after transformation might be created that results in sharp angles between connections. To reduce the presence of these angles, we propose a stitching framework to adaptively adjust border-tip segments to smooth out the gap when separated neurites are connected. The border-tip segments are defined as the segment from border-tips to the nearest branch point (shortest path from purple dot to orange dot in Figure S 2(b)). Denote $\boldsymbol{a}_{i}$ as the *i^th^* point on the tip segment of a border-tip of section *A* (*i=*1…$N_{A}$), $\boldsymbol{a}_{1}$ is the border-tip, and $\boldsymbol{a}_{N_{A}}$ is the vertex next to the branch point. To stitch border-tips $\boldsymbol{a}_{1}$ and $\boldsymbol{b}_{1}$, the shifting vector of $\boldsymbol{a}_{i}$ denoted by $\boldsymbol{v}_{i}^{a}$ is calculated as following:

| $\boldsymbol{V}_{A}=\frac{N_{A}(\boldsymbol{b}_{1}-\boldsymbol{a}_{1})}{N_{A}+N_{B}}$ | (6) |
| --- | --- |
| $\boldsymbol{v}_{i}^{a}=\frac{{(N}_{A}-i+1)}{N_{A}}\boldsymbol{V}_{A}$ | (7) |

The shifting vector of $b_{i}$ is calculated in a similar way. As illustrated in Figure S 5, the matched border-tips are moved to a new location where they are relatively close to each other while the successive points are moved by distances reduced successively until the branch point is reached. The distance to move is scaled based on the size of the tip segment such that more shifting is taken for larger segment. Notably, if there is a spine tip segment connected to a border-tip segment to stitch, the spine tip segment is moved by the shifting vector of its root as well (green dots in Figure S 5). The spine tip segments are detected by the spine filter introduced in Section 2.2.2.


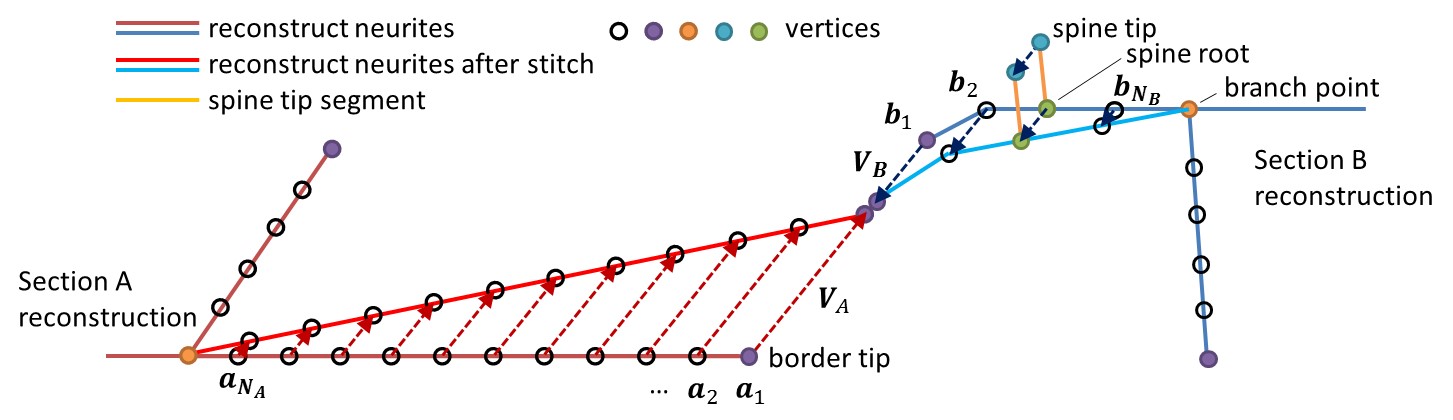


Figure S 5. Illustration of adjusting branches to stitch matched border-tips.

# Interactive User Interface

Neuron reconstruction quality will affect the performance of NeuronStitcher and the parameter selection may vary between different data. To broaden the utility of NeuronStitcher to work with a variety of different data acquisition processes, we designed an interactive interface to (1) allow visual evaluation of stitching results and live adjustments of matching parameters; and (2) enable manual correction of incorrect matching results. The software was implemented in C/C++ as a plugin of Vaa3D^17,18^, which is a publicly available open source platform with a user-friendly interface for 3D+ image analysis and visualization (<http://www.vaa3d.org>). In the following sections, we will introduce how to use the tool based on 5 scenarios. Each operation is indexed by the scenario’s ID and the step number such that A-2 means step 2 of scenario A.

## Stitch Reconstructions

In this section, we show the interactive user interface of NeuronStitcher and illustrate how to use NeuronStitcher to stitch adjacent sections. This section covers stitching neuron traces in three steps: A. Automatically match and align reconstructions, B. Visually check and stitch matched reconstructions, and C. Manually correct matching result. We then use the protocol to stitch adjacent sections.

### A. Automatically match and align reconstructions (Figure S 6, Video S 1)

A-1: Load the reconstructions to be stitched in the same 3D view in Vaa3D. Make sure the section on the bottom was loaded first. Then launch the NeuronStitcher: Plug-in->neuron_stitch-> live_stitch_neuron_SWC

A-2: Adjust the parameters in the pop-up window accordingly.

A-3: Click “Match” button. Then the program will search for border-tips, align reconstructions, and match border-tips automatically.

A-4: Check the alignment and matching result. The border-tips identified will be shown in bubbles. Matched border-tips will be in green/red colors while unmatched ones will be in dark green/magenta color. If the results are not good, adjust the parameters and repeat step A-2 and A-3.


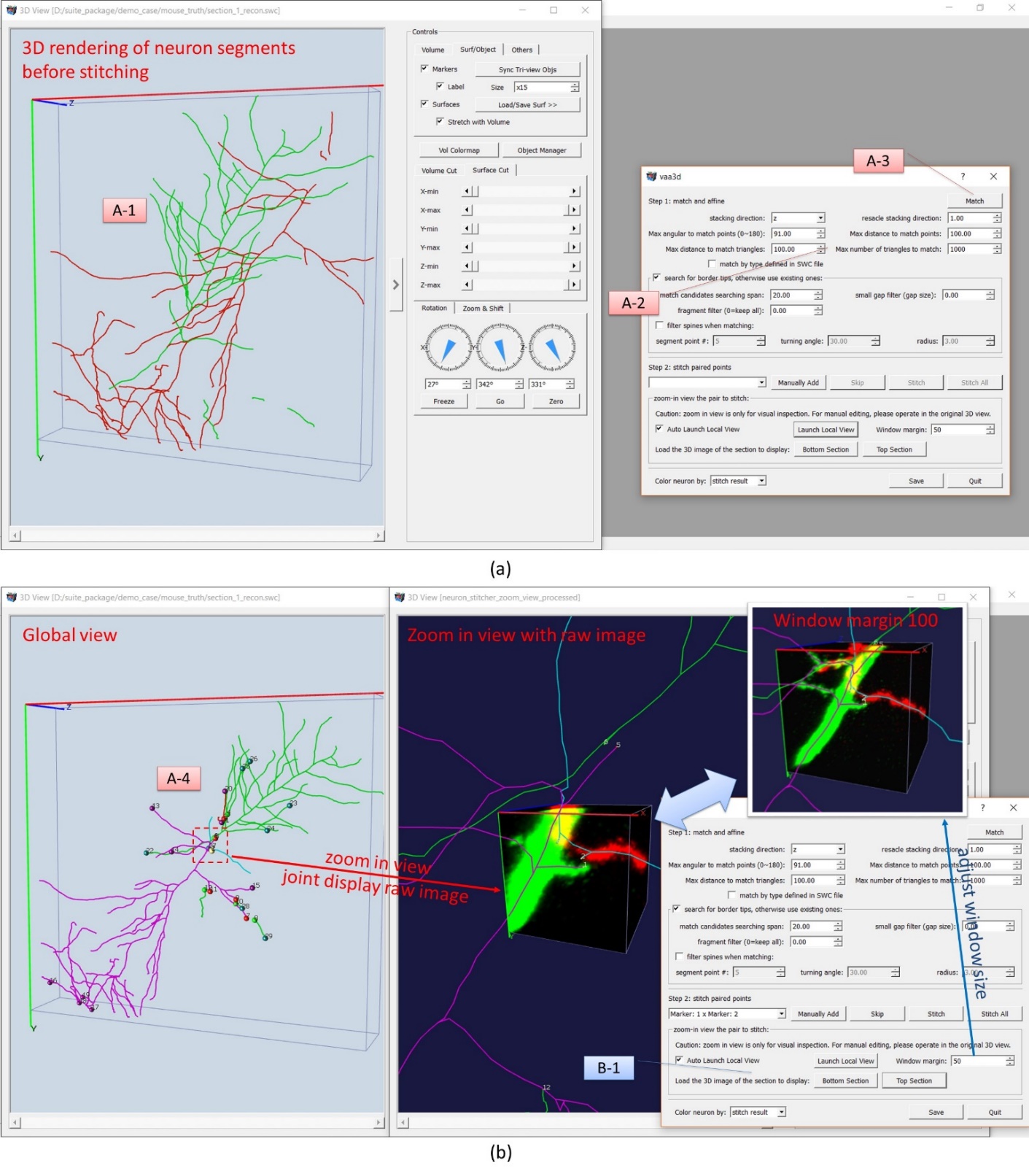


Figure S 6. Illustration of automatically matched and aligned reconstructions.

### B. Visually check and stitch matched reconstructions (Figure S 7, Video S 2-3)

B-1: A zoom-in window that focuses on the pair of matched border-tips selected will pop up by default. Clicking the “Bottom Section” or “Top Section” buttons loads the raw images of each neuron fragment for joint display in the zoom-in window (volumes are aligned into the same space and colored in red/green for the bottom/top section accordingly). Change “window margin” to adjust the size of the zoom-in window. The zoom-in window updates after clicking “Launch Local View” button or after changing the selected matched border-tips (when “Auto Launch Local View” is checked).

B-2: Visually check the pair of matched border-tips selected. The selected border-tips are shown in yellow color and the branch connected to it is highlighted in azure/magenta colors accordingly.

B-3: Click the “Stitch” button if the match is reasonable, the “Skip” button if not sure, or follow the steps in C-4 to reject and break the match if it is a mismatch. After stitching, the program jumps to the next pair of matched border-tips automatically.


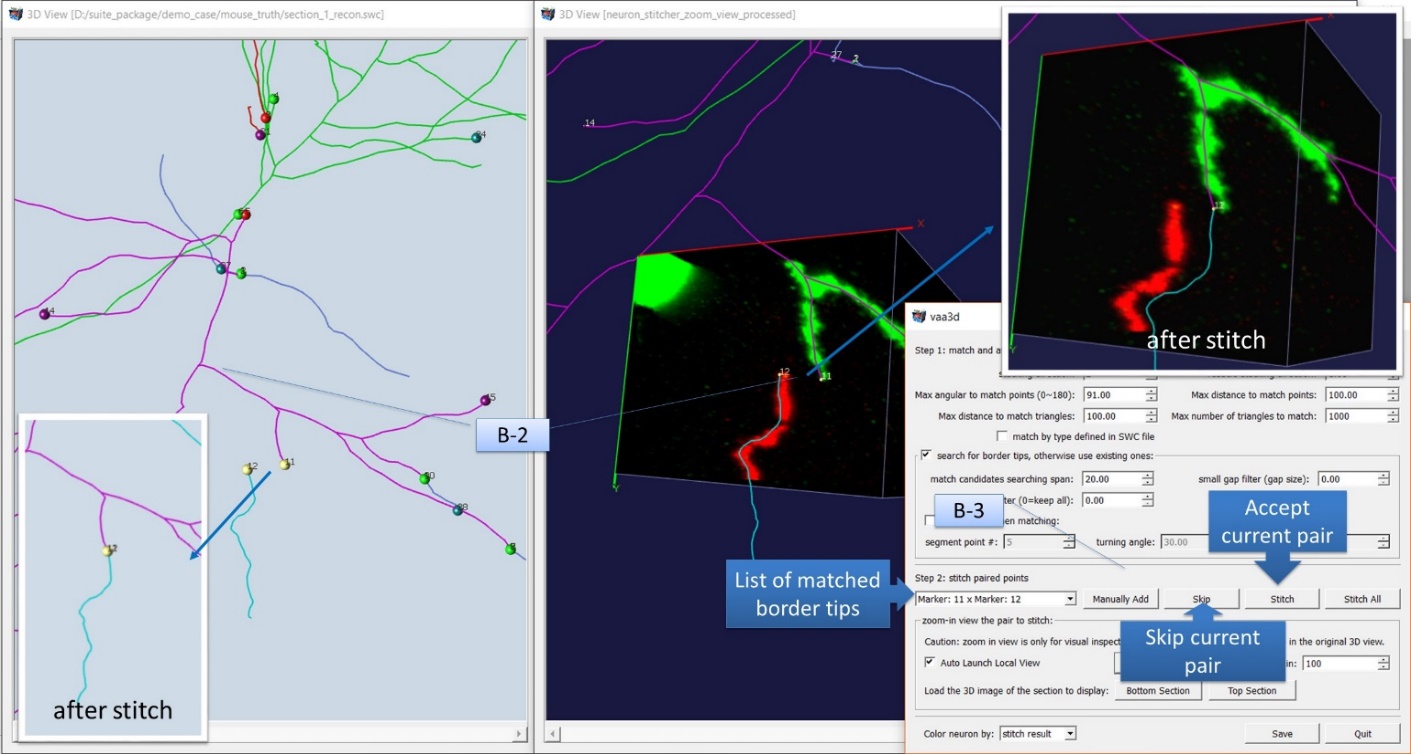


Figure S 7. Illustration of visual check and stitch matched reconstructions.

### C. Manually correct matching result (Figure S 8, Video S 4)

C-1: Identify the pair of border-tips that needs to be corrected. If there are no border-tips identified on the neurite you would like to stitch, right click on the neurite and select “create marker from the nearest neuron node” to define one.

C-2: Click the “Manually Add” button. Then a “Match Markers” dialog will pop up. On the left of the pop-up dialog is a list of matched pairs of border-tips.

C-3: To manually match two border-tips, first select the ID of the border-tips we are going to match. Then click “Match this pair of neurons” button and the matched pair will then be added to the list on the left.

C-4: To manually break a matched pair, first select the pair from the list on left. Then click “Free selected matching pair” button. The pair will then be removed from the list and the markers will be added to “Available Markers”.

C-5: Click the “Done” button after the finish. The list of matched border-tips will then be updated.


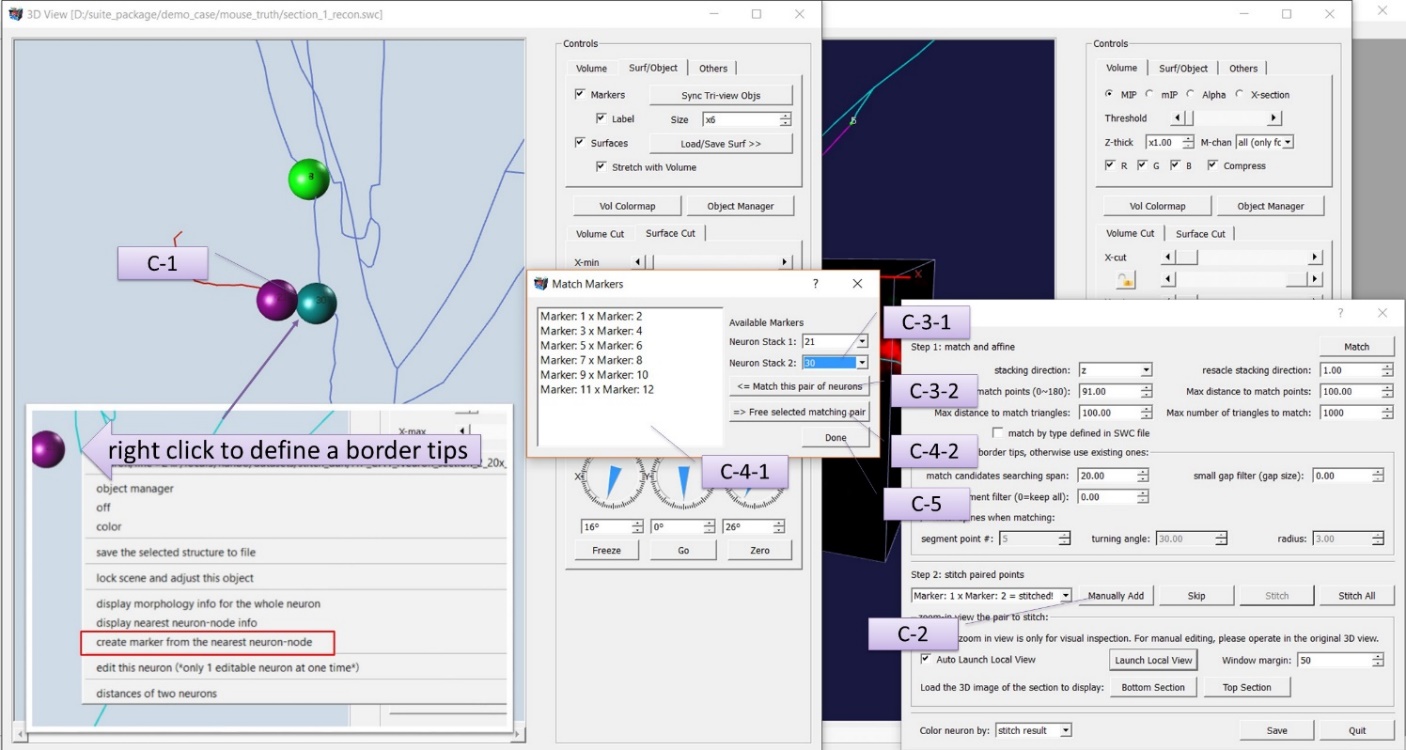


Figure S 8. Illustration of manually corrected matching result.

## Select Border-tips

### D. Select border-tips (Figure S 9)

D-1: Load the image of the neuron tissue section to search for border-tips. (Note: Loading 3D image is only for the purpose of visual inspection.)

D-2: Launch 3D view of the image by pressing ctrl+v. Load the corresponding reconstruction of neuron fragments in this section by dragging the SWC file into the 3D view.

D-3: Launch the border-tips searching tool: Plug-in->neuron_stitch->find_border_tips_SWC_image

D-4: Adjust related parameters and then click the “Search” button to automatically search for border-tips. Users can also manually define border-tips by following D-6.

D-5: The automatically found border-tips will be shown as bubbles (called Markers in Vaa3D). Visually inspect the border-tips identified one by one by following the instructions in E. Accept or reject the border-tips accordingly. The program will automatically jump to the next border-tip after acceptance or rejection.

D-6: To add a border-tip, right click on the corresponding location in 3D View and define a marker there. Then click the “Update” button in the dialog of the tool.

D-7: Click the “Save” button to save all the results. The accepted border-tips will be saved in a single file for future usage.

### E. Visually inspect border-tips (Figure S 9)

Border-tips will be shown as bubbles (called markers in Vaa3D). In 3D view, blue bubbles are the uncertain border-tips that have not been inspected yet, green bubbles are the correct border-tips that have been accepted, red bubbles are the wrong border-tips that have been rejected, and the magenta bubble is the border-tip under inspection (Figure S 9).

E-1: Global 3D view. In this view, users can inspect the global location and spatial distribution of border-tips. Missing border-tips can also be added here (D-6).

E-2: Local 3D view. In this view, users can inspect the detailed characters of the selected border-tip in a zoom in view with the focus on the border-tip. Only a local window of the 3D image will be shown in this view to allow high-resolution visualization. The size of the window is controlled by the “View Size” parameter in the tool dialog.

E-3: Slice view. The program will automatically focus on the selected border-tip in this view and highlight it by dashed line squares. In this view, users can inspect the border-tip through image slices.


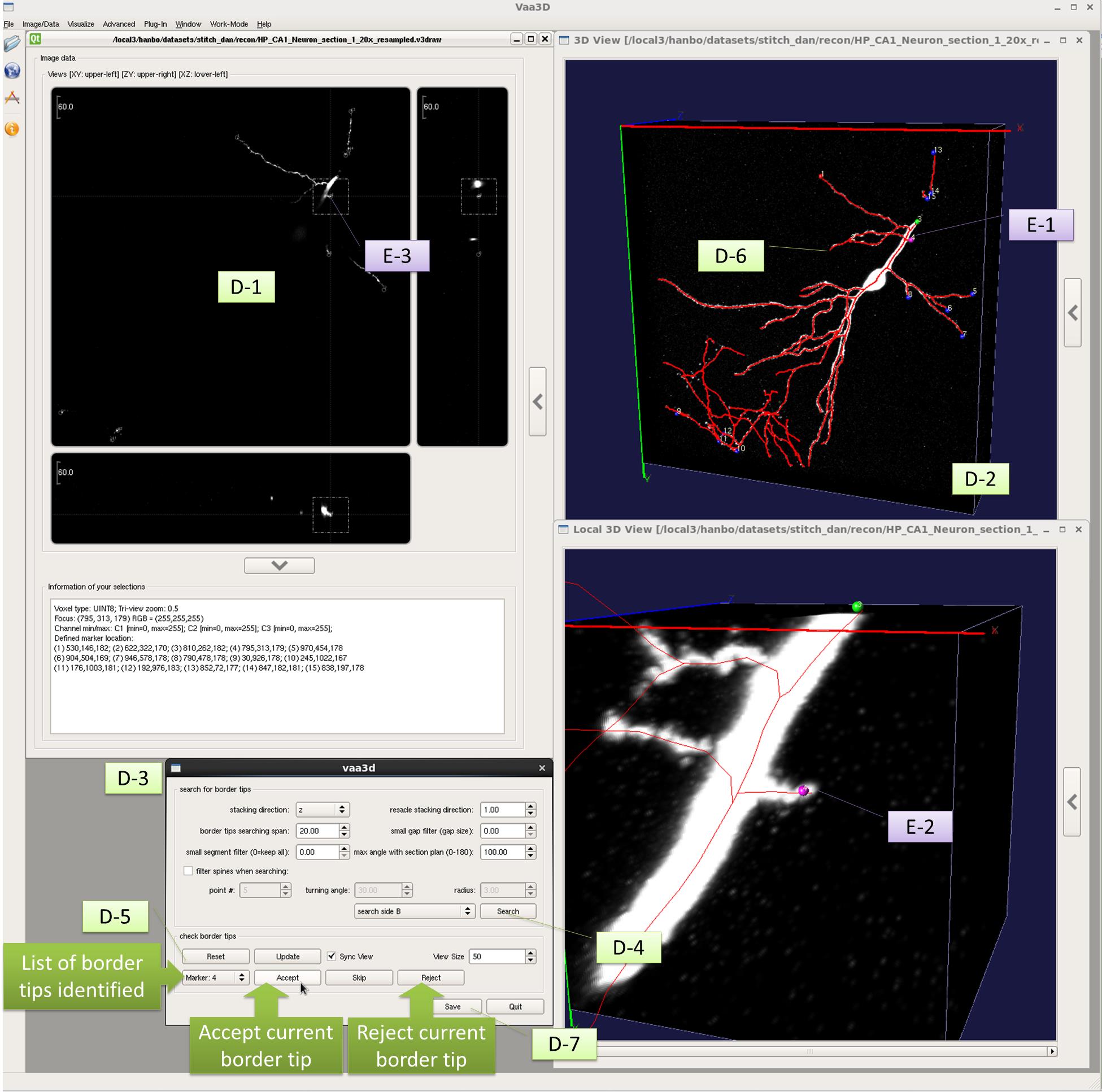


Figure S 9. Illustration of visual inspection and semi-automatic selection of border-tips.

# Results

To comprehensively evaluate the performance of NeuronStitcher, we performed tests on three types of data – 1) ground-truth data, 2) real data, and 3) simulated data. The results of each fold are shown in the following sections accordingly.

## Validation on Ground Truth Data

We evaluated the accuracy of NeuronStitcher using carefully generated “ground truth” reconstructions from a piece of mouse brain tissue containing a labeled pyramidal neuron within the hippocampal CA1 region. Sparse *in utero* electroporation was employed at embryonic day 15.5 in CD1 mice to target the developing hippocampus. The lateral ventricles of the embryos were injected with 1 µg/µl of TdTomato plasmid in phosphate buffered saline (PBS) with 10 % Fast Green dye for visualization. The embryos were electroporated using four pulses of 42V for 50 ms at 500 ms intervals. Afterward, the uterus was placed back in the abdominal cavity and the incision was sutured.

At postnatal day 14, mice were perfused with 4% paraformaldehyde in PBS and brains were incubated overnight in 4% paraformaldehyde at 4° C. After 3 washes with PBS, serial 600 µm vibratome sections were cut and mounted on slides with imaging spacers and Vectashield mounting media. This sample was first imaged with a 10x 0.45 NA objective lens and the neuron was thereafter semi-automatically reconstructed in 3D by Vaa3D and manually corrected by an expert as the ground truth reconstruction (Figure S 10(a)).

After imaging, tissue sections were removed from the microscope slide, embedded in 3% agarose in PBS, and sectioned with a vibratome at 200 µm intervals. The tissue sections were reimaged with a 20x 0.75 NA objective lens. The reconstructions of neuron fragments from all such individual sections were generated by an expert with a similar approach and then stitched together using NeuronStitcher (Figure S 10(b-c)).

Using the automatic matching module, 6 pairs of matched border-tips were identified between section #1 and #2 (Figure S 10(d)) and 2 pairs of matched border-tips were identified between section #2 and #3 (Figure S 10(e)). A careful comparison of the ground truth reconstruction to the stitched reconstruction showed that 98% bifurcations of the 3D reconstructed, tree-like neuron morphology in the ground truth had their correspondence in the stitched reconstruction, and the minor amount (2%) of missing correspondence occurred at the section interfaces and was due to the sectioning process. Notably, the thinner 200 µm tissue sections reduced the signal loss resulting from light scattering from imaging through deep tissues. As a result, some deep neurites missing from the original reconstruction were captured after reimaging the 200 µm tissue sections (highlighted by yellow circles in Figure S 10(f)).


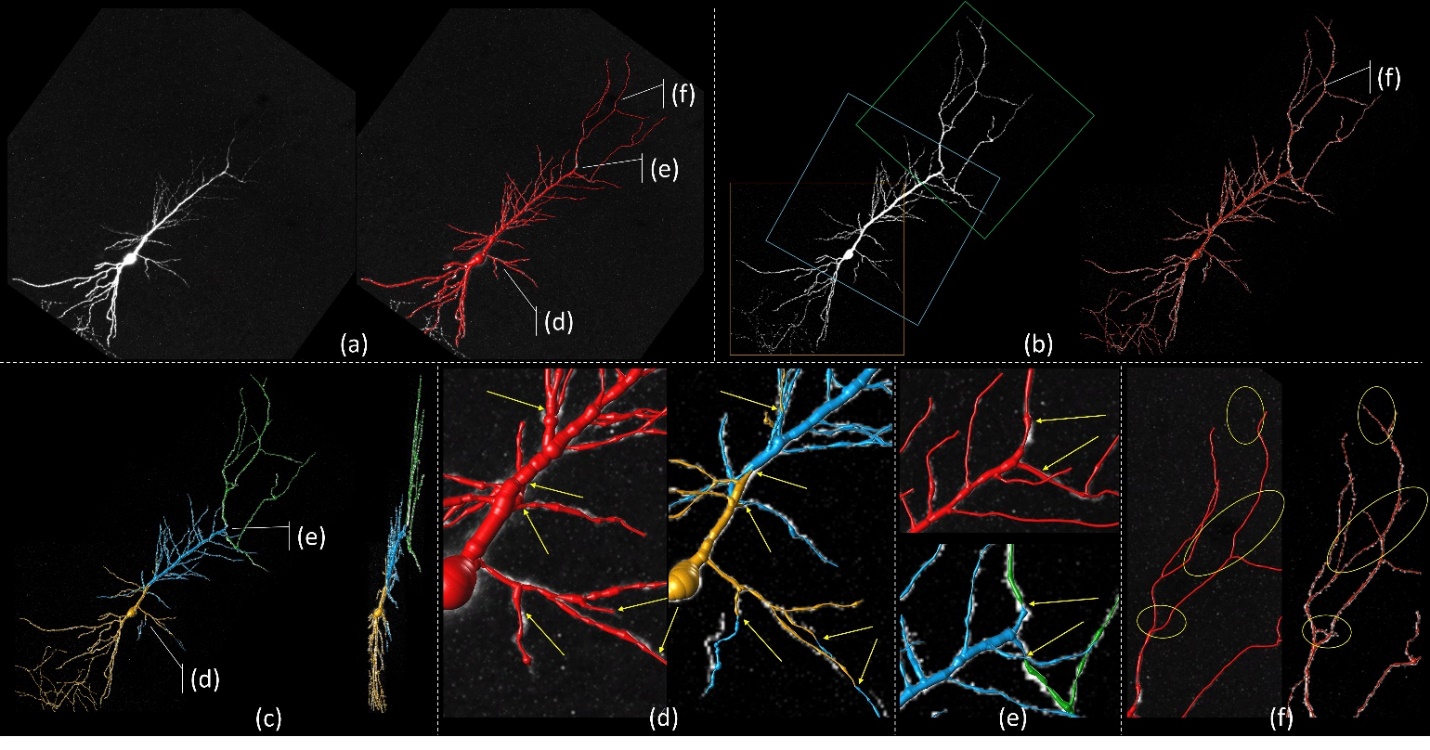


Figure S 10. A visual comparison between the reconstruction from the tissue before sectioning and the stitched reconstruction from sectioned tissues. (a) Maximum projection of 3D image acquired from complete tissue (left) and the corresponding reconstruction (right). The image has been rotated for better comparison with the stitching result. (b) Automatically aligned and stitched reconstructions from 3 successive sectioned tissues (right). The images of tissue sections were also aligned based on the stitching result and the section boundaries are highlighted by colored boxes accordingly (left). (c) Stitched reconstructions color-coded by sections (yellow: #1, blue: #2, green: #3) from different view angles. (d)-(f) Zoomed in view of regions shown in (a)-(c). The ground truth reconstruction is shown on the top/left of each subfigure. The stitched reconstruction is shown on the bottom/right of each subfigure. The matched and stitched border-tips and their corresponding locations on original reconstruction are highlighted by yellow arrows.

## Large Scale Neuron Image in Mammalian Brain

We used three sets of large-scale microscopy images obtained from mouse or human brains and their corresponding neuron reconstructions to test the performance of our neuron stitching framework (Table S 2). Dataset 1 was obtained from mouse brain V1 area and contains 11 adjacent sections in which the neurites of two neurons were densely twisted (Figure S 11). Dataset 2 contains a single mouse V1 neuron imaged in 4 adjacent sections. The reconstructions in dataset 2 were automatically generated using the all-path-pruning 2 (APP2) algorithm^21^ and spine segments were also reconstructed (Figure S 13). Dataset 3 contains 2 neurons from a human brain tissue imaged by bright field microscopy.

Table S 2. Information of data applied for stitching test cases.

| ID | Species | Data Description | Imaging Method | # of Sections | x,y,z Resolution (μm) | Reconstruction Process |
| --- | --- | --- | --- | --- | --- | --- |
| 1 | Mouse | Two adjacent neurons in V1. | Confocal | 11 | 0.14,0.14,0.28 | Neuron Crawler^22^, APP2^21^, Manual Correction |
| 2 | Mouse | A single neuron in V1, border of layer 3 and 4. | Confocal | 4 | 0.14,0.14,0.28 | Neuron Crawler^22^, APP2^21^ |
| 3 | Human | Two biocytin-filled neurons. | Bright  field | 4 | 0.11,0.11,0.28 | Adaptive Enhancement^23^, Neuron Crawler^22^, APP2^21^ |

We stitched each pair of adjacent sections using NeuronStitcher. After loading the reconstruction, we performed automatic matching and alignment. Based on visual inspections, we adjusted the parameters to obtain satisfactory matching results. In total, 16 pairs of sections were stitched. 1504 border-tips between those section pairs were identified. Among them, 411 pairs of border-tips were automatically matched. After automatic matching, an expert manually corrected the errors by using our interactive proof-reading module when necessary. 356 (86.6%) pairs were accepted and 55 pairs were rejected by the expert. In addition to the automatically matched border-tips, other 59 pairs of border-tips were manually matched (Table S 3). NeuronStitcher typically finished the computation within seconds and the memory required for computation is less than 100Mb. The time to visually check and adjust the results depended on the complexity of the reconstruction. For the datasets here, the average time for stitching (including the time of automated computation, visual inspection, parameter fine-tuning, and manually adjustment of the result) an adjacent pair of serial sections was 13’08” (median: 9’41”, minimum: 0’13”, and maximum: 36’31”) (Table S 3). The final results are visualized with each section in different colors in Figure S 11-16.

Table S 3. Stitching results between sections based on the live stitching module: labor time is the time that experts took to adjust parameters, visually check matching and alignment results, and manually adjust matching results.

| Data ID | Section ID | | Border-tips Identified | | Automatic Matched Pairs | Labor Time | Accepted Pairs | Rejected Pairs | Manually Matched Pairs |
| --- | --- | --- | --- | --- | --- | --- | --- | --- | --- |
|  | A | B | A | B |  |  |  |  |  |
| 1 | 1 | 2 | 3 | 3 | 3 | 0'13" | 3 | 0 | 0 |
| 1 | 2 | 3 | 14 | 15 | 11 | 2'20" | 8 | 3 | 1 |
| 1 | 3 | 4 | 33 | 55 | 26 | 7' | 23 | 3 | 5 |
| 1 | 4 | 5 | 81 | 47 | 41 | 31'50" | 36 | 5 | 5 |
| 1 | 5 | 6 | 75 | 71 | 53 | 31'23" | 45 | 8 | 6 |
| 1 | 6 | 7 | 72 | 71 | 43 | 22'47" | 33 | 10 | 9 |
| 1 | 7 | 8 | 64 | 95 | 45 | 36'31" | 36 | 9 | 5 |
| 1 | 8 | 9 | 39 | 34 | 27 | 14'47" | 22 | 5 | 11 |
| 1 | 9 | 10 | 25 | 33 | 23 | 6'03" | 20 | 3 | 4 |
| 1 | 10 | 11 | 23 | 23 | 19 | 3'09" | 19 | 0 | 1 |
| 2 | 1 | 2 | 10 | 29 | 8 | 4'00" | 6 | 2 | 2 |
| 2 | 2 | 3 | 45 | 85 | 22 | 11'45" | 20 | 2 | 4 |
| 2 | 3 | 4 | 59 | 82 | 21 | 14'07 | 19 | 2 | 1 |
| 3 | 1 | 2 | 17 | 43 | 13 | 4'00" | 11 | 2 | 2 |
| 3 | 2 | 3 | 44 | 69 | 23 | 7'36" | 22 | 1 | 2 |
| 3 | 3 | 4 | 85 | 60 | 33 | 12'44" | 33 | 0 | 1 |


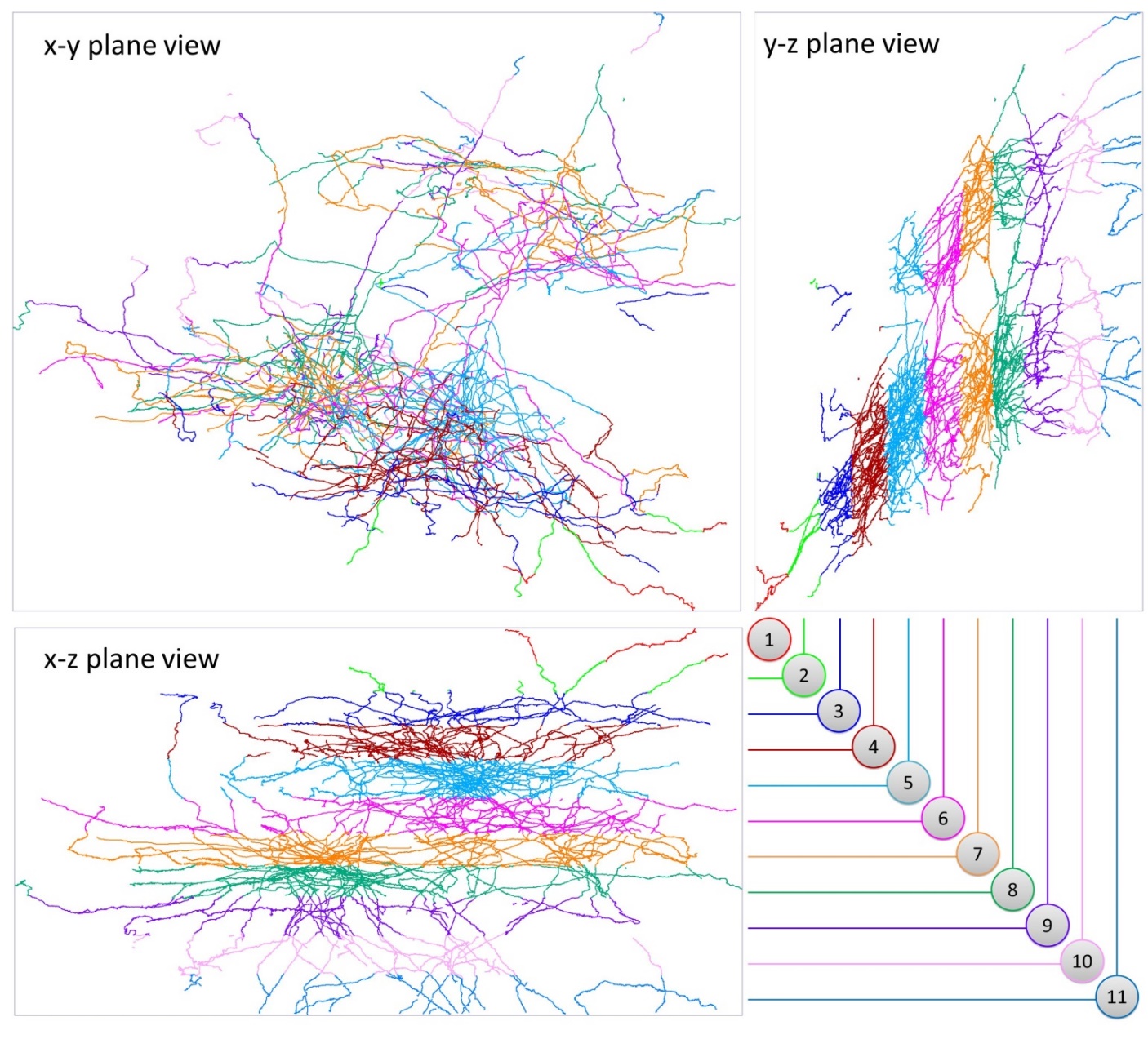


Figure S 11. Visualization of stitching results of dataset 1 from different views: reconstructions are displayed in skeleton; segments are in different colors for each section.


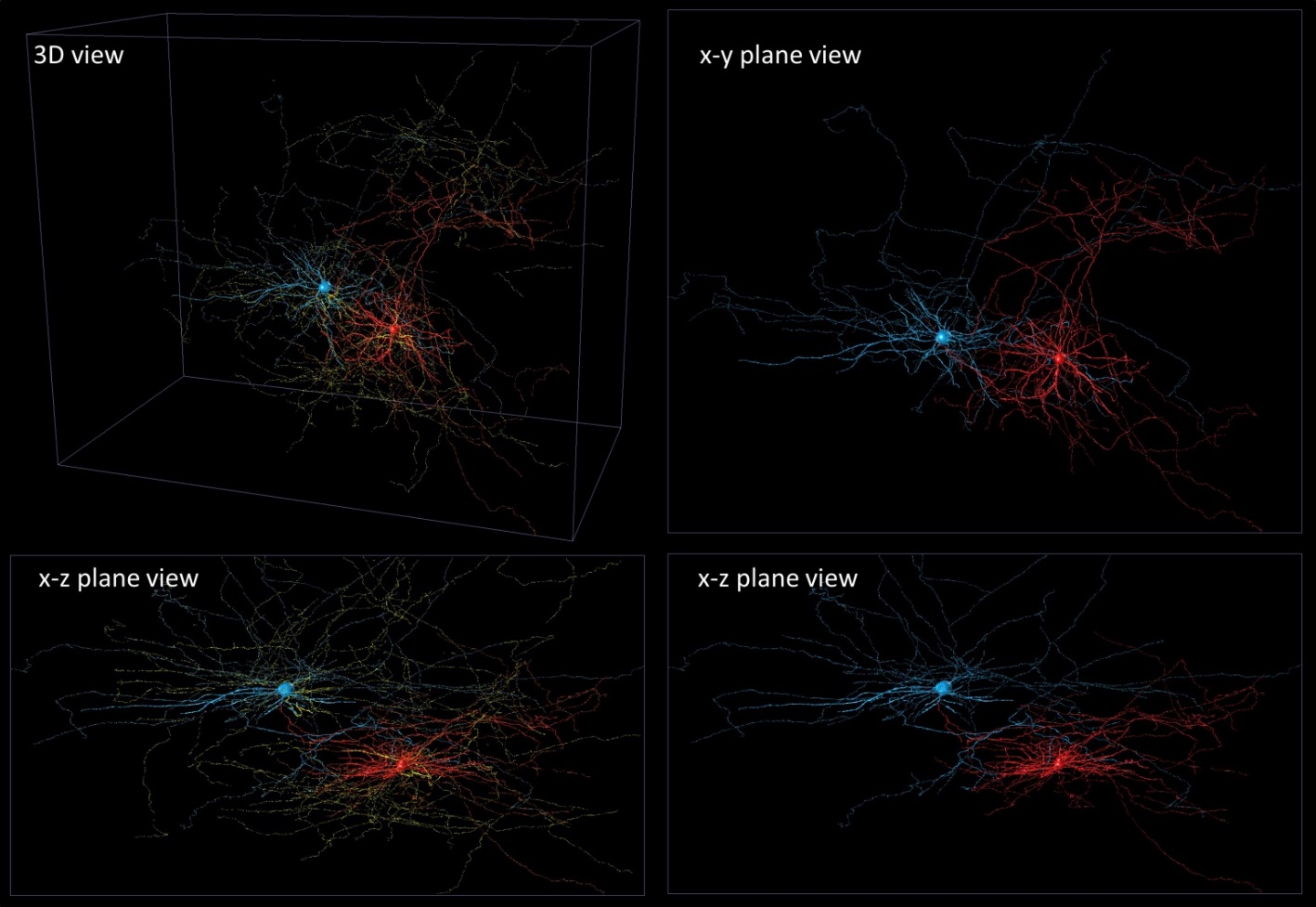


Figure S 12. Visualization of complete neuron reconstruction of dataset 1 after stitching. Reconstructions are displayed in surface mode. Two neurons are shown in different colors (red and blue). Yellow branches are the rest segments that were not yet connected to the somata and are removed in the sub-figures on the right.


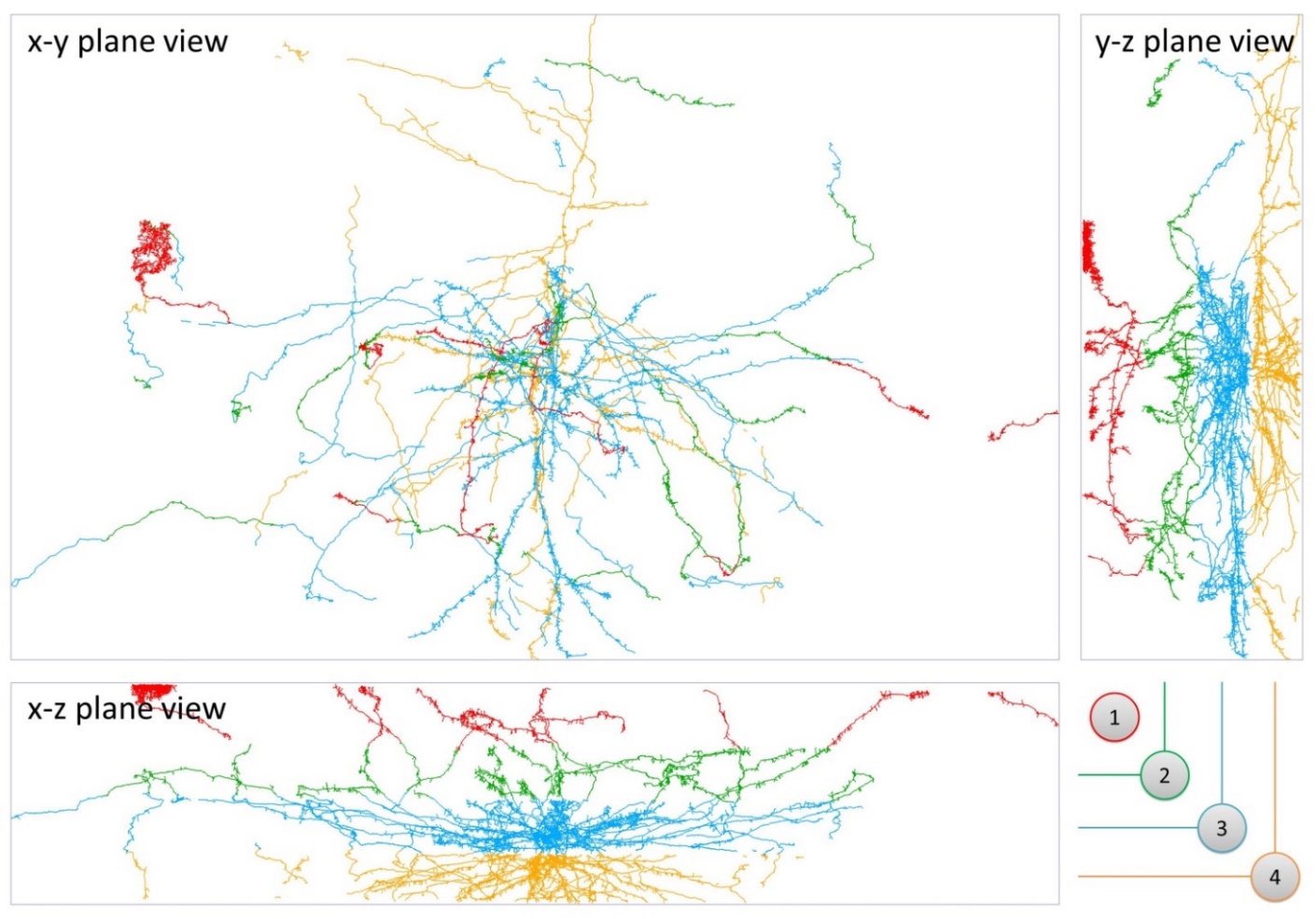


Figure S 13. Visualization of stitching results of dataset 2 from different views. Reconstructions are displayed in skeleton. Segments are in different colors for each section.


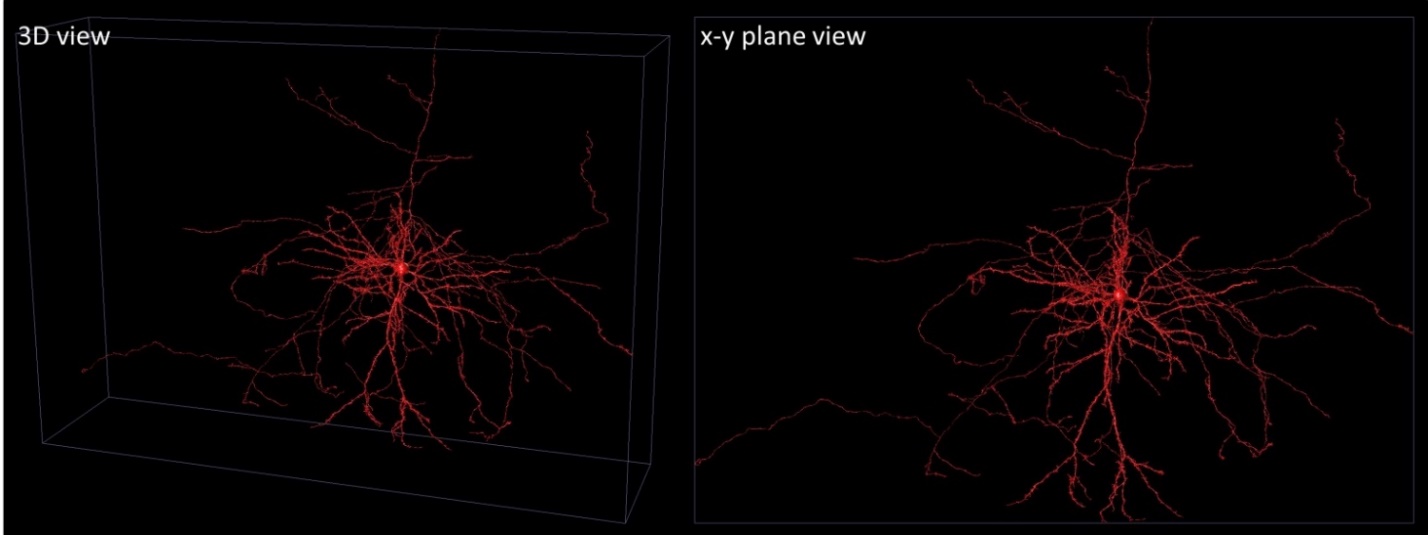


Figure S 14. Visualization of the complete neuron reconstruction of dataset 2 after stitching; Reconstructions are displayed in surface mode. Only branches connected to the soma were extracted for visualization.


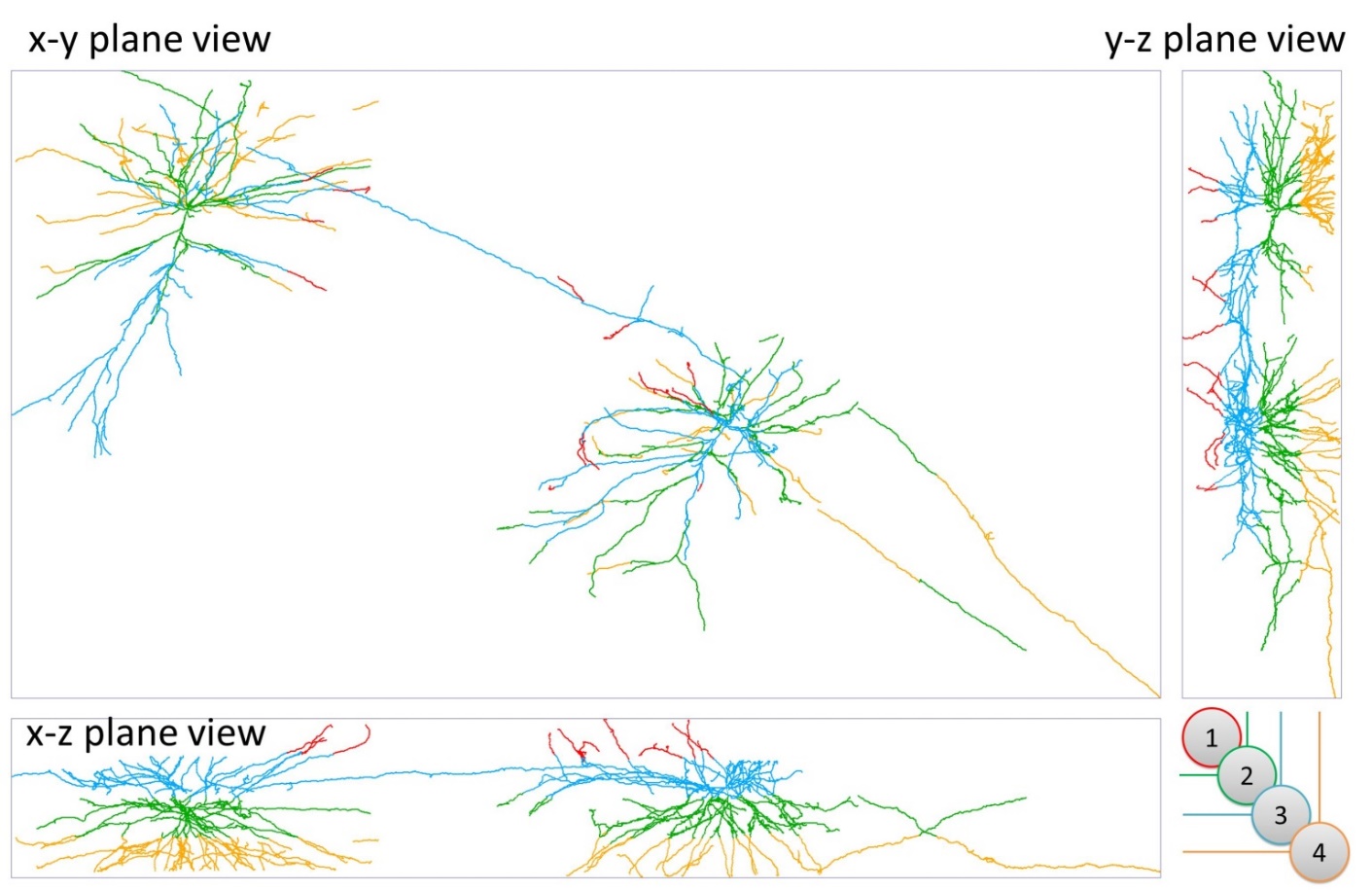


Figure S 15. Visualization of stitching results of dataset 3 from different views. Reconstructions are displayed in skeleton. Segments are in different colors for each section.


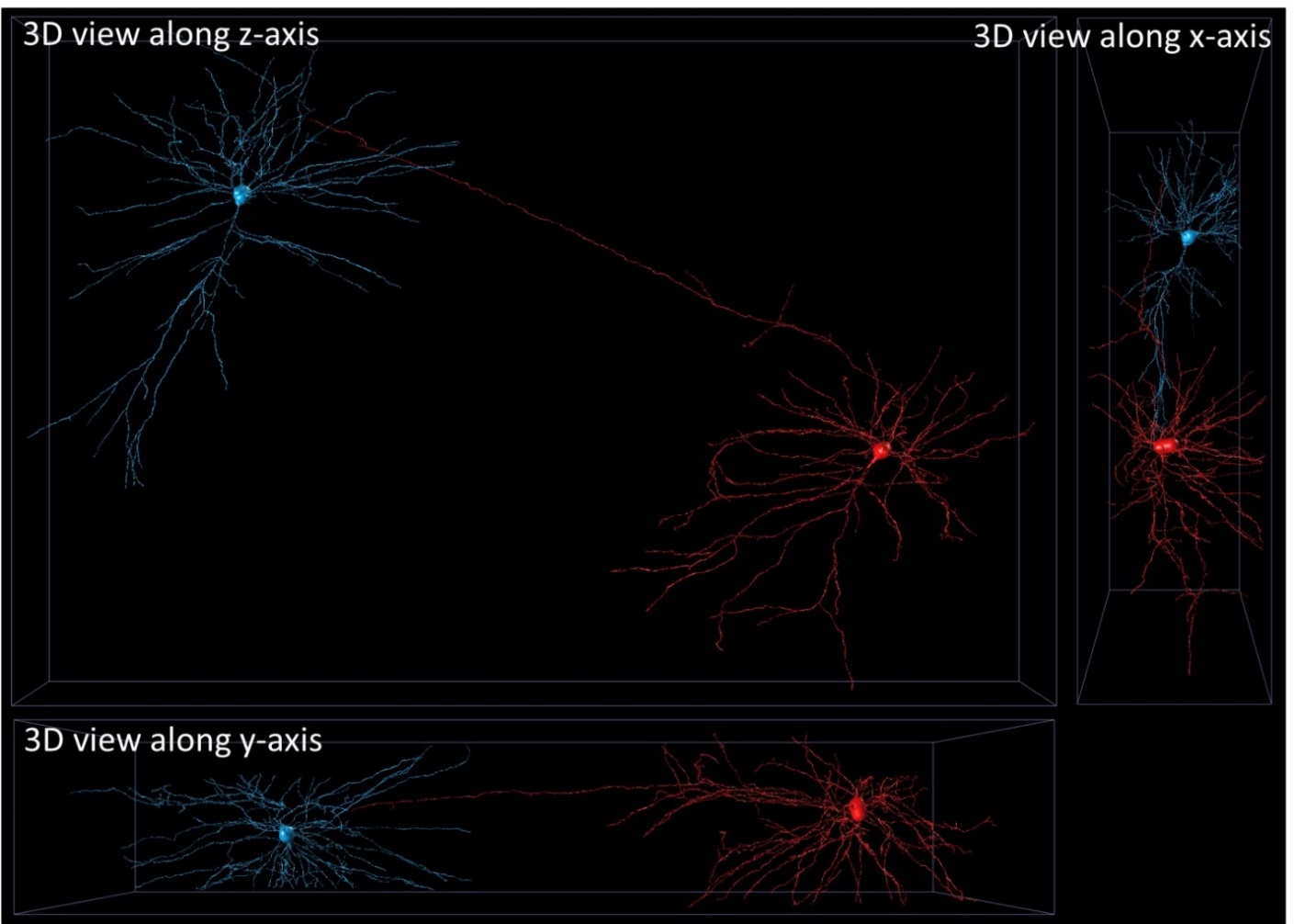


Figure S 16. Visualization of the complete neuron reconstruction of dataset 3 after stitching. Reconstructions are displayed in surface mode. Only branches connected to the somata were extracted for visualization. Two neurons are shown in different colors.


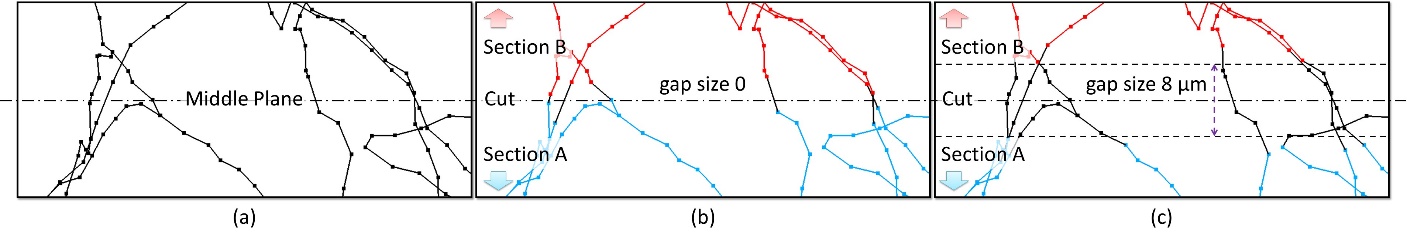


Figure S 17. Illustration of the generation of simulated data from experimental data. (a) Reconstruction of experimental data. (b) Simulated data with gap size 0. (c) Simulated data with gap size 8 μm. In (b) and (c), black lines are the removed components after separation. Red and blue lines are the sections after separation.

## Simulated Testing Data

We also considered an alternative way to produce “ground truth” to evaluate the accuracy of NeuronStitcher. We chose 5 densely arborized reconstructions from the mouse visual cortex (dataset 1 previously used) and digitally “sectioned” each of them into two halves by deleting the vertices and the edges severed by the gap (black vertices and edges in Figure S 17) to generate the simulated data. Several different gap configurations (1, 2, 4, and 8 μm) were used to simulate the different levels of tissue loss during sectioning. One half was randomly rotated and shifted in parallel to the sectioning plane to make those sections dis-aligned. An example of simulated data when the gap size is 8 μm is shown in Figure S 18(b).


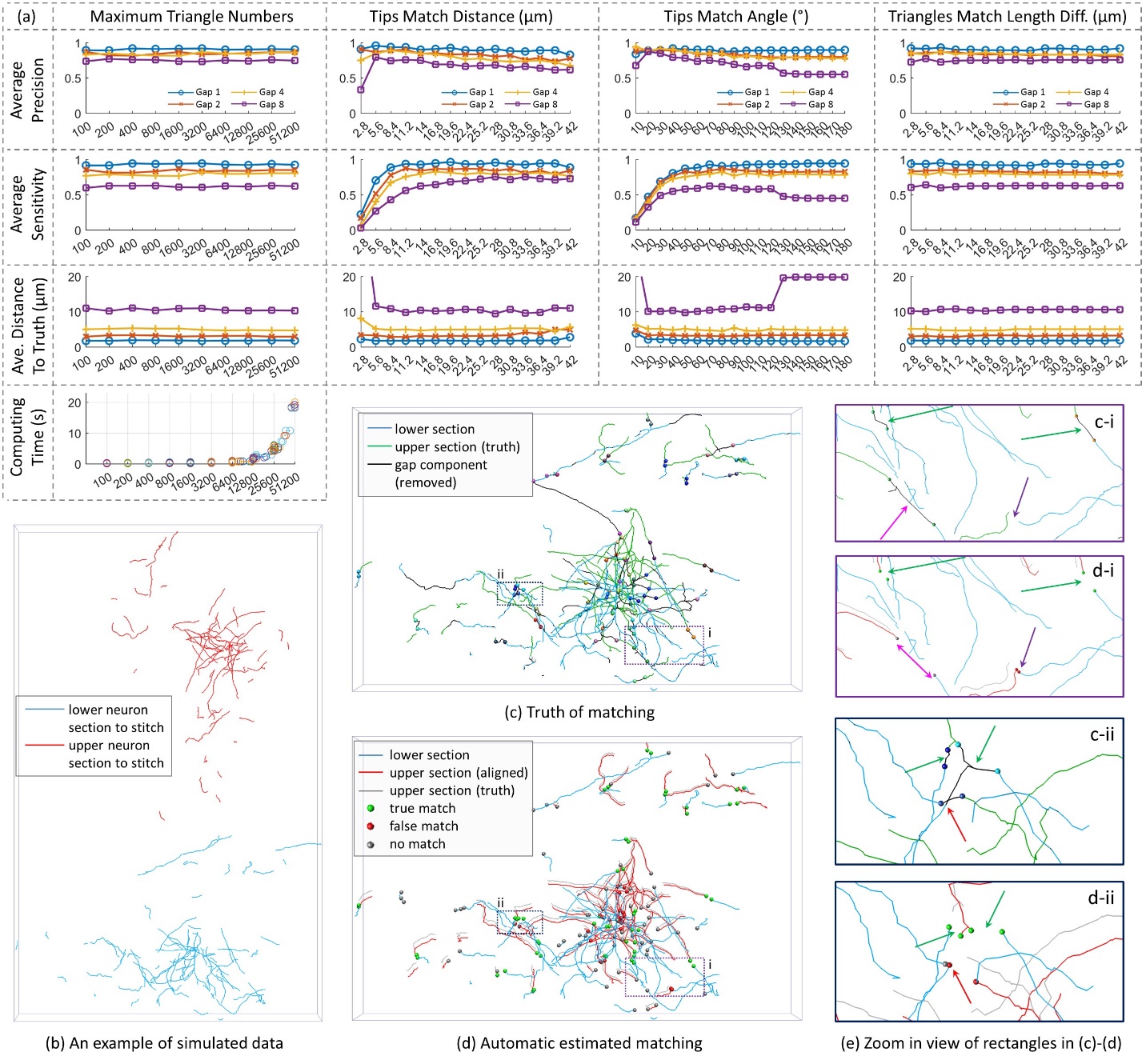


Figure S 18. The performance of automatic matching module on simulated data. (a) Quantitative assessment of performance with different parameter configurations. Each column corresponds to the tuning of a specific parameter (the parameter name is on the top) and each row corresponds to a specific measurement (the measurement name is on the left). In each subfigure, the x-axis is the value of selected parameter and the y-axis is the performance measurement. The default values for the parameters are: maximum triangle number: 6400, distance to match tips: 14μm (100 voxels), angle to match tips: 70°, length difference to match triangles: 14μm (100 voxels). (b)-(e) An example of (b) simulated data (test case 2, gap size 8 μm),(c) truth data, and (d)-(e) matching and alignment result. In (e), green arrows highlighted correctly matched border-tips, magenta arrows highlighted the miss-matched border-tips (false negative), purple arrows highlighted the matching of none-border-tips (false positive), and red arrows highlighted falsely matched border-tips.

Then, we applied NeuronStitcher to the simulated data to automatically stitch them back together based on different parameter configurations. An example of automatic matching of simulated data is shown in Figure S 18(b-e). As shown in Figure S 18(d), even when a considerable amount of tissue is lost (black curves in Figure S 18(c)), the automatically estimated alignment is relatively close to the ground truth and most of the severed neurites were correctly matched. However, falsely matched border-tips can still be observed. As highlighted by red and purple errors in Figure S 18(e), some branches are falsely connected when the neurites are densely distributed in a small area. And some connections are missed when a long branch in parallel to the sectioning plane is severed (magenta errors in Figure S 18(e)). To quantify the performance of proposed matching algorithm, we compared the result to the ground truth in the following two aspects:

1) Spatial distance:

Because the topology structure of section B remains the same when moving it in the 3D space, we measured the average distance between corresponding vertices before moving (ground truth) and after alignment (results by proposed algorithm). When the distance was relatively small, the proposed algorithm estimated the correct alignment.

2) Topology correctness:

The proposed algorithm matched tips between section A and section B. After matching, we expected the vertices that were separated after separation (ground truth) to be reconnected (matched) and the vertices that were not connected at the beginning to remain disconnected. Thus, based on ground truth, precision and sensitivity defined below were measured:

| $Precision=\frac{\left\Vert{Match}^{*}\cap{Match}^{truth} \right\Vert}{\left\Vert{Match}^{*} \right\Vert}$ | (8) |
| --- | --- |
| $Sensitivity=\frac{\left\Vert{Match}^{*}\cap{Match}^{truth} \right\Vert}{\left\Vert{Match}^{truth} \right\Vert}$ | (9) |

where *Match^*^* is the set of matched pairs of border-tips obtained by the proposed algorithm and *Match^truth^* is the set of pairs of vertices separated during separation. If a branch (fork vertices) was removed during separation that resulted in 3 or more vertices separated at a time, matching any pair of these vertices was counted as a successful match.

For different gaps, by using the default parameter configuration, the average precision in matching border-tips gradually changed from 90% to 75% while the average sensitivity was between 93% and 62% - indicating the alignment result was accurate enough to recapitulate the original reconstruction. Though the performance of our matching algorithm is potentially affected by the quality of neuron reconstruction, the results suggested that it is still robust enough to generate correct alignment when there is a considerable amount of information loss (gap size 8 μm).

We then adjusted 4 critical parameters to examine the robustness of the proposed method. These 4 parameters and their default values were: maximum triangle number: 6400, distance to match tips: 14μm (100 voxels), angle to match tips: 70°, length difference to match triangles: 14μm (100 voxels). During each test, only 1 parameter was adjusted while the other 3 were fixed to the default value. It is evident that our proposed method is robust to various parameter combinations. The distance between the final alignment and the “ground truth” are reasonably good among most parameter selections. This suggests that our alignment algorithm is robust to the parameter selection. In practice, such robustness is critical to reducing the time of parameter tuning.

One potential concern of our proposed algorithm is that by sampling triangles, we may lose information and thus cannot achieve a good alignment. However, as shown in Figure S 18(a), the parameter of number of triangles used for matching did not significantly affect the final matching result. Notably, by only taking the first 100 triangles, the obtained results were similar to the results of using all triangles while the computational time was reduced from 20 seconds to less than 1 second. Because the triangle match is designed to vote for the best initial transformation and the later process will iteratively refine the matching and alignment to achieve the best result, as long as a correct initial matching can be identified, the similar final results will be obtained. And this result suggested that our triangle sampling scheme efficiently picked the correct information to estimate initial border-tips matches.

On the other hand, the parameters used in matching refinement procedure (the maximum distance and angle to match border-tips) have more impact on the accuracy of final results. For instance, the purple line (gap 8) in the 3^rd^ column of Figure S 18(a) has a sudden jump between 120° and 130°. This is because the objective function of our matching is to generate as many matched pairs as possible. And we do not penalize distance or direction dis-similarity between matched border-tips. Thus when we release the constraint on direction similarity by increasing angle threshold from 120° to 130°, the algorithm found another solution which though will give more matched border-tips but is less correct. Also, in the 2^nd^ and 3^rd^ column of Figure S 18(a), we can see a tradeoff between precision and sensitivity. Intuitively, if either the distance or angle criterion is too restrictive, a lot of severed neurites will be missed for matching. Conversely, with looser criteria, more false connections will be generated. In practice, we suggest the users selecting the parameter that can (1) generate correct alignment and (2) find as many matches as possible (trade in precision to achieve high sensitivity). Since our tool allows users to proofread and correct all the matched border-tips in later steps, they can easily reject falsely matched border-tips then.

# Comparison

In comparison with the previous works^7,12,13,15^, NeuronStitcher has four advantages over other methods. (1) NeuronStitcher includes a filter to eliminate noise resulting from terminal branches based on three criteria when selecting severed neurite candidates. Notably, the accuracy of reconstruction matching largely relies on the identification of severed neurites. Filtering noisy objects before analysis improves the matching accuracy and computational speed. (2) The performance of NeuronStitcher has been intensively and quantitatively evaluated with different types of data. In most previous works, the performance of the automatic matching algorithm is usually evaluated by a few simple cases without quantification. In this paper, our proposed algorithm has been quantitatively evaluated by ground truth data, complicated real problem data of different types, and synthetic data. Specifically, with synthetic data, we quantitatively examined the performance of our proposed automatic stitching algorithm and showed that the algorithm is robust to both information loss and parameter selections. (3) Our method has an interactive interface which not only allows visual evaluation of stitching results and live adjustments of matching parameters but also enables manual correction of matching results. (4) Our method is readily available to other researchers. The software was implemented in C/C++ as a plugin of Vaa3D^17,18^, which is a publicly available Open Source platform with a user-friendly interface for 3D+ image analysis and visualization (<http://www.vaa3d.org>).

For quantitative comparison, we tested the performance of Filament Editor on our simulated dataset. After testing, we found that the performance of Filament Editor is sensitive to two parameters – “Max Point Distance” and “Alpha”. To further quantify and compare its performance, we picked 5 values for “Max Point Distance” (20,50,100,200,300) and 4 values for “Alpha” (0.01,0.05,0.1,0.2) and used different combinations of parameters to match the 20 synthetic data we previously generated. Then the precision, sensitivity of matching and the distance to the “ground truth” are measured in the similar way as we did in the previous section. As shown in Figure S 19, Filament Editor’s performance is similar to NeuronStitcher when there is relatively low information loss (e.g. 1 μm gap). However, when the amount of information loss increases, Filament Editor’s performance degrades. Specifically, when the gap size is 8 μm, NeuronStitcher can still achieve 70% precision, 58% sensitivity, and 12.3 μm distance to the “ground truth” on average for different parameter configurations. In comparison, Filament Editor failed to estimate correct matching on test case 1 with 8 μm gap. For the remaining cases, its performance fluctuated dramatically by different parameters. And even if we picked the best parameter combinations, it still cannot achieve similar performance as NeuronStitcher on test case 2 and 5.


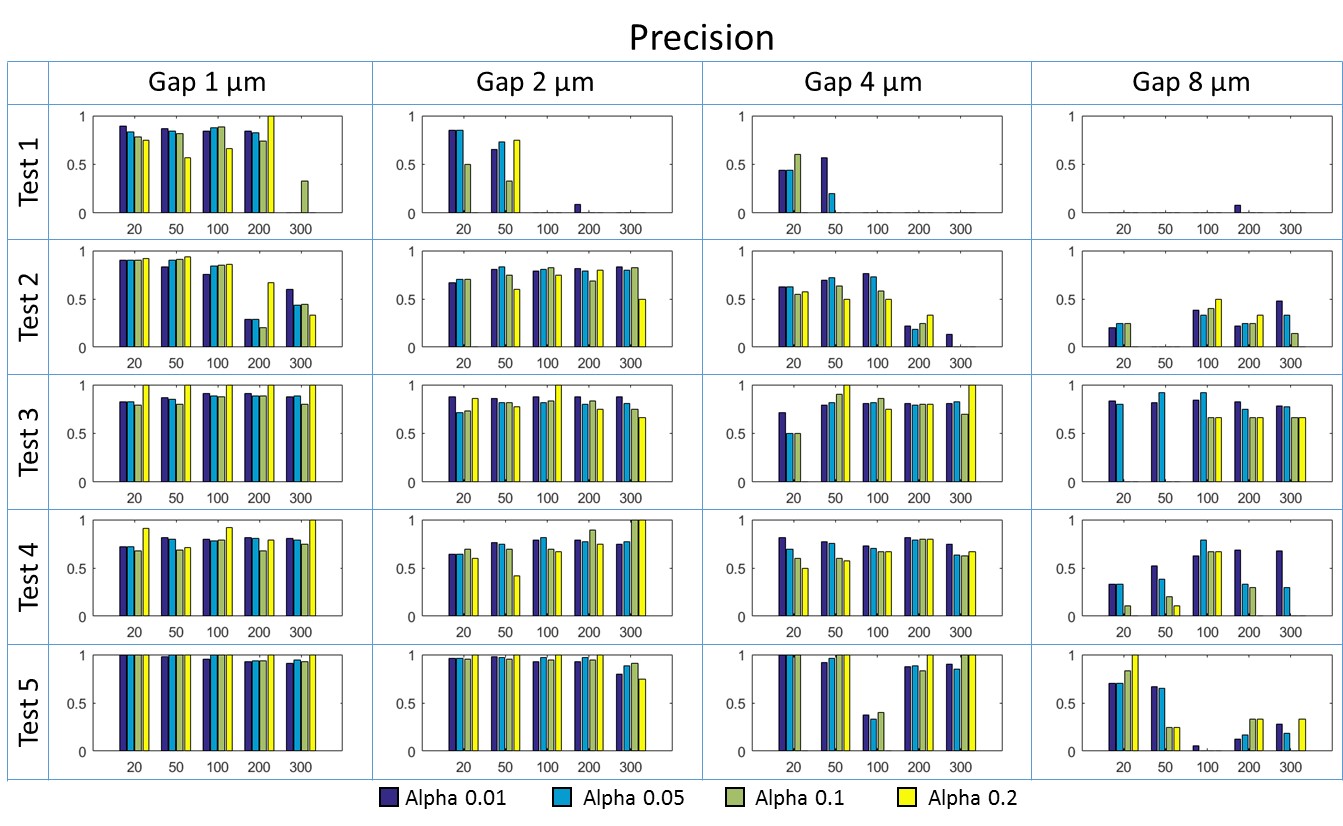


(a)


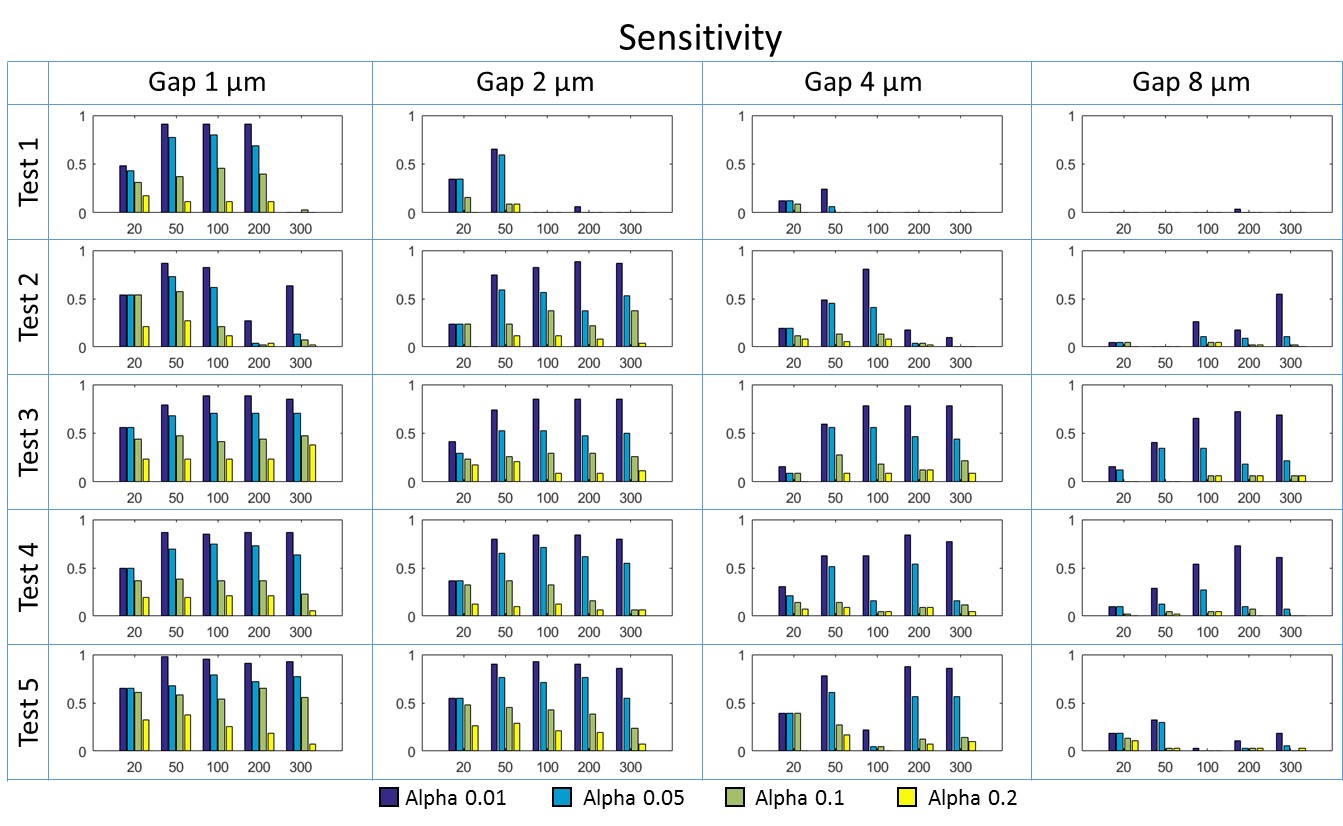
(b)


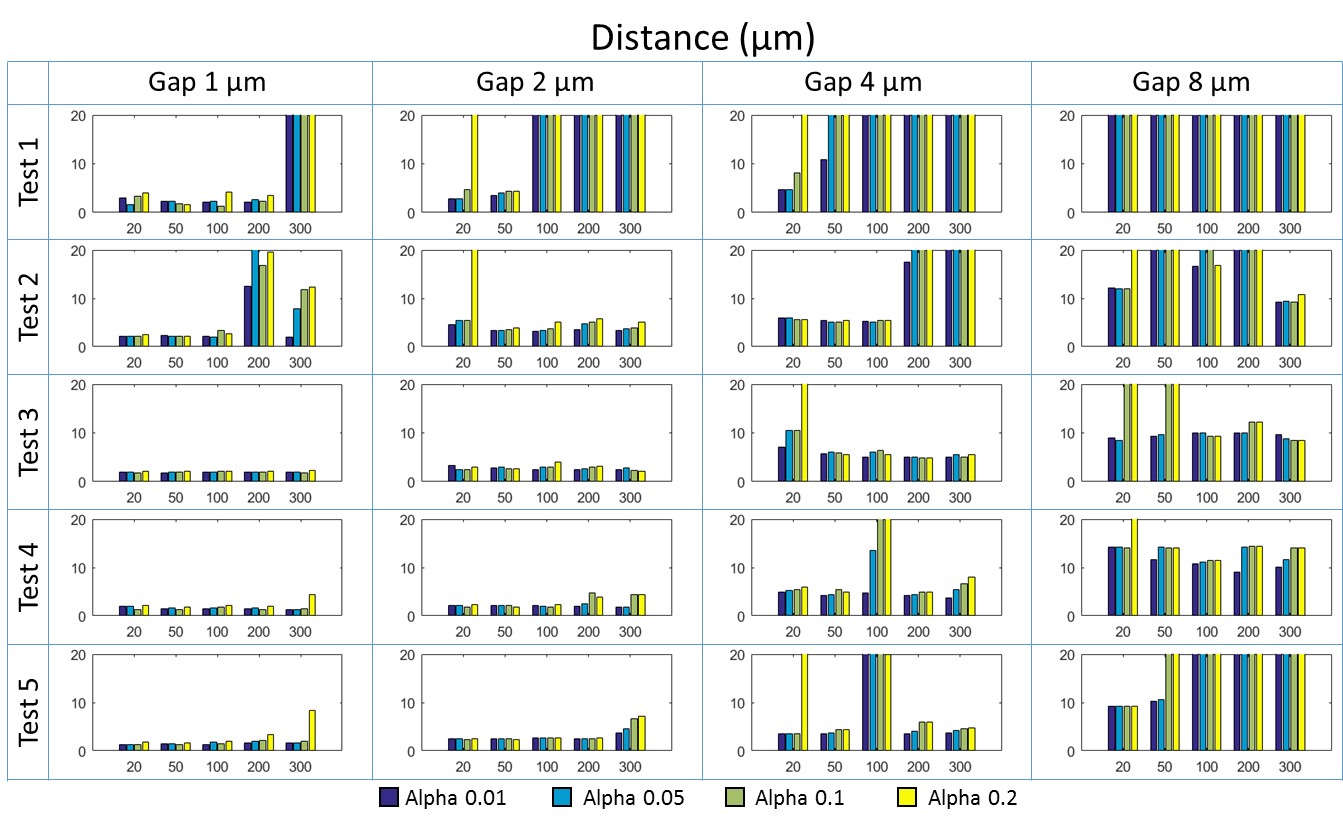
(c)

Figure S 19. The performance of Filament Editor in matching and aligning neuron fragments. The same 20 synthetic data that we previously generated were used here. The respective y-axis in (a) (b) (c) are the precision, sensitivity, and distance. The x-axis corresponds to five different values of “Max Point Distance”. Bars in four different colors correspond to four different “Alpha” values. In (c), to make the figure easier to plot, the real distance values are displayed as “20um” when they are greater than 20 μm.

Despite the low robustness to information loss, the Filament Editor simply takes all the neuron reconstruction terminations close to the sectioning plane for matching. Thus its performance could be sensitive to the noise border-tips we introduced in previous sections. And unlike NeuronStitcher, the Filament Editor does not automatically generate a final stitching result. It requires users to manually connect aligned fragments, which is inconvenient and unfriendly particularly for situations of dense and complex fragments. Moreover, the commercial nature of Filament Editor prevents the free use of the software. Of note, both NeuronStitcher and Vaa3D is Open Source, free, and readily available.

# Reference

1. Helmstaedter, M. & Mitra, P. P. Computational methods and challenges for large-scale circuit mapping. *Curr. Opin. Neurobiol.* **22,** 162–9 (2012).

2. Parekh, R. & Ascoli, G. A. Neuronal morphology goes digital: a research hub for cellular and system neuroscience. *Neuron* **77,** 1017–38 (2013).

3. Meijering, E. Neuron tracing in perspective. *Cytometry. A* **77,** 693–704 (2010).

4. Chung, K. *et al.* Structural and molecular interrogation of intact biological systems. *Nature* **497,** 332–7 (2013).

5. Hama, H. *et al.* Scale: a chemical approach for fluorescence imaging and reconstruction of transparent mouse brain. *Nat. Neurosci.* **14,** 1481–8 (2011).

6. Oberlaender, M., Bruno, R. M., Sakmann, B. & Broser, P. J. Transmitted light brightfield mosaic microscopy for three-dimensional tracing of single neuron morphology. *J. Biomed. Opt.* **12,** 64029 (2007).

7. Luzzati, F., Fasolo, A. & Peretto, P. Combining confocal laser scanning microscopy with serial section reconstruction in the study of adult neurogenesis. *Front. Neurosci.* **5,** 70 (2011).

8. Preibisch, S., Saalfeld, S. & Tomancak, P. Globally optimal stitching of tiled 3D microscopic image acquisitions. *Bioinformatics* **25,** 1463–5 (2009).

9. Bria, A. & Iannello, G. TeraStitcher - a tool for fast automatic 3D-stitching of teravoxel-sized microscopy images. *BMC Bioinformatics* **13,** 316 (2012).

10. Cardona, A. *et al.* TrakEM2 software for neural circuit reconstruction. *PLoS One* **7,** e38011 (2012).

11. Helmstaedter, M., Briggman, K. L. & Denk, W. High-accuracy neurite reconstruction for high-throughput neuroanatomy. *Nat. Neurosci.* **14,** 1081–8 (2011).

12. Dercksen, V. J., Hege, H.-C. & Oberlaender, M. The Filament Editor: an interactive software environment for visualization, proof-editing and analysis of 3D neuron morphology. *Neuroinformatics* **12,** 325–39 (2014).

13. Weber, B. *et al.* Automated stitching of microtubule centerlines across serial electron tomograms. *PLoS One* **9,** e113222 (2014).

14. Dercksen, V. J. *et al.* Automatic alignment of stacks of filament data. in *2009 IEEE International Symposium on Biomedical Imaging: From Nano to Macro* 971–974 (IEEE, 2009). doi:10.1109/ISBI.2009.5193216

15. Hogrebe, L. *et al.* Trace driven registration of neuron confocal microscopy stacks. in *2011 IEEE International Symposium on Biomedical Imaging: From Nano to Macro* 1345–1348 (IEEE, 2011). doi:10.1109/ISBI.2011.5872649

16. Donohue, D. E. & Ascoli, G. A. Automated reconstruction of neuronal morphology: an overview. *Brain Res. Rev.* **67,** 94–102 (2011).

17. Peng, H., Ruan, Z., Long, F., Simpson, J. H. & Myers, E. W. V3D enables real-time 3D visualization and quantitative analysis of large-scale biological image data sets. *Nat. Biotechnol.* **28,** 348–53 (2010).

18. Peng, H., Bria, A., Zhou, Z., Iannello, G. & Long, F. Extensible visualization and analysis for multidimensional images using Vaa3D. *Nat. Protoc.* **9,** 193–208 (2014).

19. Shen, D., Wong, W. & Ip, H. H. S. Affine-invariant image retrieval by correspondence matching of shapes. *Image Vis. Comput.* **17,** 489–499 (1999).

20. Duchenne, O., Bach, F., Kweon, I.-S. & Ponce, J. A Tensor-Based Algorithm for High-Order Graph Matching. *IEEE Trans. Pattern Anal. Mach. Intell.* **33,** 2383–2395 (2011).

21. Xiao, H. & Peng, H. APP2: automatic tracing of 3D neuron morphology based on hierarchical pruning of a gray-weighted image distance-tree. *Bioinformatics* **29,** 1448–54 (2013).

22. Zhou, Z., Sorensen, S. A. & Peng, H. Neuron crawler: An automatic tracing algorithm for very large neuron images. in *2015 IEEE 12th International Symposium on Biomedical Imaging (ISBI)* 870–874 (IEEE, 2015). doi:10.1109/ISBI.2015.7164009

23. Zhou, Z., Sorensen, S., Zeng, H., Hawrylycz, M. & Peng, H. Adaptive Image Enhancement for Tracing 3D Morphologies of Neurons and Brain Vasculatures. *Neuroinformatics* **13,** 153–166 (2015).
